# The Emergence of Extracellular Electron Mediating Functionality in Rice Straw-Artificial Soil Mixture during Humification

**DOI:** 10.3390/ijerph192215173

**Published:** 2022-11-17

**Authors:** Tingting Hu, Duyen Minh Pham, Takuya Kasai, Arata Katayama

**Affiliations:** 1 Graduate School of Engineering, Nagoya University, Tokai National Higher Education and Research System, Nagoya 464-8603, Japan; 2 Institute of Materials and Systems for Sustainability, Nagoya University, Tokai National Higher Education and Research System, Nagoya 464-8603, Japan

**Keywords:** EEM material-dependent dechlorinating anaerobic consortium, organic matter decomposition, quinone synthesis, specific electric capacitance, rice straw, humification

## Abstract

This study aimed to elucidate the origin of extracellular electron mediating (EEM) functionality and redox-active center(s) in humic substances, where they are ubiquitously distributed. Here, we show the emergence of EEM functionality during the humification of rice straw in artificial soil (kaolin and sand) with a matric potential of −100 cm at 20 °C for one year. We used the dechlorination activity of an EEM material-dependent pentachlorophenol-dechlorinating anaerobic microbial consortium as an index of the EEM functionality. Although rice straw and its mixture with artificial soil did not initially have EEM functionality, it emerged after one month of humification and increased until six months after which the functionality was maintained for one year. Chemical and electrochemical characterizations demonstrated that the emergence and increase in EEM functionality were correlated with the degradation of rice straw, formation of quinone structures, a decrease in aromatic structures, an increase in nitrogenous and aliphatic structures, and specific electric capacitance during humification. The newly formed quinone structure was suggested as a potential redox-active center for the EEM functionality. These findings provide novel insights into the dynamic changes in EEM functionality during the humification of organic materials.

## 1. Introduction

Humic substances are natural organic macromolecules formed by the humification process and are ubiquitous in soil, sediments, and natural water bodies [1]. Classical definition of humification relies on the synthesis of large molecules from decomposition products of biotic debris of plants, animals, and micro-organisms and has been explained by various hypotheses, such as lignin, polyphenol, and sugar-amine condensation theories, based on the extensive study of the chemical structures of humic substances [1,2,3,4]. However, the emergent view of the humification process entails a continuum model from large debris to a smaller molecular size, involved with formation/destruction of aggregates and adsorption/desorption toward mineral surfaces [3].

The humification process has been studied with a focus on changes in the chemical structures of organic molecules [1,5,6,7]. Over the last three decades, both soluble and insoluble humic substances have been reported to have extracellular electron mediating (EEM) functionality in various microbial reactions, including dissimilatory iron reduction, contaminants detoxification of halogenated organic compounds, nitroaromatics, azo dyes, and pharmaceuticals [8,9,10,11,12,13,14,15,16,17,18,19,20]. The EEM functionality of humic substances has drawn more attention because of its importance in microbial reactions of biogeochemical cycles of carbon, nitrogen and other elements [15,16,21,22,23,24] and its applicability to bioelectrochemical systems for bioremediation [25,26]. The ubiquitous EEM functionality distribution in humic substances raises the question of its origin. Plant biomass-derived black carbon (biochar), formed by incomplete combustion of biomass, has been widely reported to have EEM functionality. Both biochars and humic substances are ubiquitously distributed in soil and sediments and have the same quinone/semiquinone moieties as redox-active centers [27,28]. Nitrogenous and sulfurous (non-quinone) functional groups have also been reported to be responsible for the redox mediating capacity of humic substances [29,30]. In addition, the humification process of biowaste composting has been extensively studied [31], as it is an environmentally friendly technology to treat biowaste. The increase in EEM functionality of humic acids during the composting of various biowaste has been reported [32]. Thus, we hypothesized that the ubiquitously distributed EEM functionality in humic substances originated during the humification process.

Laboratory incubation of organic matter has previously been extensively studied to model its decomposition/humification process in soil [33,34]. Study on EEM functionality during the humification process has been mainly focused on compost humification instead of laboratory incubation, although the end-product of compost humification was different from that of soil [35]. On one hand, the temperature of laboratory incubation study for organic matter decomposition was commonly set up in the range of 5–35 °C [36], whereas the composting process reached up to 70 °C during the thermophilic phase. This could result in a difference in the microbial community during both humification processes [37,38]. In addition, clay/minerals were widely involved in laboratory incubation study [39,40], as they were the major inorganic fraction of soil [41], but not in compost humification. Mineral binding has been reported as a major mechanism for organic matter stabilization and mineral colloids also catalyze abiotic humification [42,43]. However, the change in EEM functionality during the humification of laboratory incubation study with clay/minerals has not been documented until now.

Humification of rice straw, a typical lignocellulosic organic material, has been extensively studied as it is a ubiquitous agricultural waste worldwide [44,45,46,47]. Research on the decomposition of rice straw in soil has concentrated on the kinetic mineralization process and dynamical change in the chemical structures of organic carbon, especially with the involvement of the radioisotope ^14^C [45]. Carbon in rice straw is either mineralized to CO_2_/CH_4_ or transformed to microbial products during humification, affected by environmental factors (i.e., temperature, moisture content, and oxygen accessibility) and its own properties (i.e., composition, C/N ratio) [48,49,50]. Rice straw has a complex and rigid microstructure with three main components: cellulose, hemicellulose, and lignin. Cellulose and hemicellulose are easily attacked by cellulolytic organisms and decompose more rapidly than aromatic polymer lignin [51]. However, the study on changes in the electrochemical property of rice straw during humification was limited.

Thus, this study aims to elucidate changes in EEM functionality and electrochemical properties, along with changes in the chemical structures during the humification of rice straw in laboratory aerobic incubation. Here, we demonstrated the emergence and increase in EEM functionality in a rice straw-artificial soil mixture for one year of humification with chemical and electrochemical characterization.

## 2. Materials and Methods

### 2.1. Rice Straw-Artificial Soil Mixtures and Humification Conditions

Rice straw (Oryza sativa variety Aichi-no-Kaori) obtained from the Nagoya University farm (Aichi, Aichi, Japan) was air-dried, pulverized using a Wonder crusher WC-3 (Osaka Chemical Co., Osaka, Japan), and sieved (<300 μm) prior to use in the experiment. The elemental composition of the sieved rice straw powder is shown in Table S1. Artificial soil was composed of kaolin (Kanto Chemical Co., Inc., Tokyo, Japan) and industrial quartz sand (No.7, 53–212 μm of size distribution, Mikawa Keiseki Co., Ltd., Okazaki, Aichi, Japan), (1:2, w/w), without any organic matter, modified from OECD guideline [52]. Rice straw powder (5 g) and artificial soil (45 g) were placed in a glass bottle (ф = 55 mm), mixed well, moistened with distilled water at matric potential −100 cm and then mixed well again. The ratio of rice straw to artificial soil, 10%, was selected to provide a high organic carbon content (4%) in the general range from 0.73 to 4.8% of organic carbon content of top 20 cm in crop land in the world [53]. The moistened mixture was inoculated with 1 mL of Kamajima paddy soil suspension supernatant (1:10, w/w) obtained after two hours of standing. The inoculated rice straw-artificial soil mixture is abbreviated as Mix. The glass bottle containing Mix was covered with aluminum foil and incubated for one year in the dark at 20 °C for humification. Water was added weekly to compensate for water loss by evaporation. The artificial soil with inoculation (no rice straw) was also prepared as a control (AS) and incubated under the same conditions. Triplicate samples were tested after 0, 1, 3, 6 months, and 1 year of humification and labeled as 0M, 1M, 3M, 6M, and 1Y, respectively. The samples were freeze-dried, ground, and stored in a freezer (−30 °C) until subjected to the following experiments. Triplicate samples of Mix with different humification periods were named by combining the abbreviations of material-period-replication; for example, Mix-1M-1 (the rice straw-artificial soil mixture humified for one month, replication 1). The composite of the three replicates was named Mix-1M-C. The humification experiment of Mix was repeated under the same conditions to verify the results.

### 2.2. Evaluation of EEM Functionality Using an EEM Material-Dependent Pentachlorophenol (PCP) Dechlorinating Anaerobic Consortium

The EEM functionality of the sample was evaluated using an anaerobic humin-dependent PCP-to-phenol dechlorinating consortium [10,54,55]. The PCP dechlorinating consortium was maintained by 5% (*v*/*v*) transfer in a medium containing 40 g/L of humin, extracted as described by Pham and Katayama (2018) [56]. The humin concentration was determined prior to the experiment by selecting the culture with the highest dechlorination activity under the conditions with different humin concentrations (15 g/L, 30 g/L and 40 g/L) (Figure S1). This PCP-dechlorinating consortium requires humin as an extracellular electron mediator for dechlorination activity [10]. This microbial consortium cannot use H_2_ and acetate as electron donors for microbial dechlorination [54], although they were widely accepted as electron donors for anaerobic dechlorinators [57]. Therefore, this consortium is called the EEM material-dependent PCP-dechlorinating anaerobic consortium. Dechlorination activity was used to evaluate the EEM functionality of the samples by replacing humin in the medium.

The EEM functionality of the sample was evaluated by placing 1 g of the sample in a 20 mL N_2_-bubbled mineral medium supplemented with trace minerals and sealed with a Teflon-coated butyl rubber stopper and aluminum seal. The medium containing the sample was bubbled again with N_2_ gas for 40 min, and the headspace was flushed with N_2_ gas for 20 min and then autoclaved (121 °C, 20 min). The autoclaved medium was supplemented with sodium formate (10 mM as the final concentration), PCP sodium salt (20 μM), and 0.2 mL filter-sterilized vitamin solution [12,14]. Then, the medium was inoculated with the EEM material-dependent PCP-dechlorinating culture 5% (*v*/*v*) and incubated in the dark at 30 °C for 21 days. This was regarded as the first generation. The EEM functionality as a PCP-dechlorination activity was evaluated using the third generation obtained by repeating a 5% (*v*/*v*) transfer of the culture to a new medium containing the same sample. Using a third generation enabled us to avoid the effects of substances carried over from the original humin-containing culture. The EEM functional intensity of the sample was evaluated based on the metabolite composition of the PCP dechlorination. When PCP was dechlorinated to 3-chlorophenol (3-CP) and phenol, and no PCP remained, it was judged to have strong EEM activity. In contrast, EEM was considered inactive when PCP was not dechlorinated, or 2, 3, 4, 5-tetrachlorophenol (2, 3, 4, 5-TeCP) was detected as the sole metabolite of PCP (excluding phenol), and the amount was less than 10% (mol/mol). The activity between these two was defined as intermediate activity. The activity was regarded as increasing when fewer substituted chlorophenols were detected or when their proportions increased. The mole number of chorine (Cl) removed from one mole of PCP, N_Cl_ (dimensionless number), was also used as index to indicate the EEM functionality, which was calculated as the sum of the products of numbers of Cl removed from individual metabolites and their proportions to the total amount of PCP and metabolites detected after the incubation (Calculation S1). For the evaluation, a medium containing 40 g/L humin was used as a positive control. Abiotic controls (negative control-a) consisted of media containing 1 g of the Mix samples with different humification periods without inoculation. No organic matter controls (negative control-b) were provided using media containing 1 g of AS with different humification periods and inoculations. Inoculated rice straw without humification (no artificial soil) was used as a negative control-c.

PCP and its metabolites in the cultures were extracted with an acetonitrile and toluene mixture (1:1, *v*/*v*) and analyzed using a QP-2010 gas chromatography-mass spectrometer (Shimadzu, Kyoto, Japan) equipped with a DB-5MS column (J&W Scientific Inc., Folsom, CA, USA) [58], or by an LC-20 high-performance liquid chromatography (LC) (Shimadzu, Kyoto, Japan) equipped with an InertSustain AQ-C18 column (3 μm particle size, 2.1 mm in inner diameter, 150 mm length, GL Sciences Inc., Tokyo, Japan) and an SPD-M20A photodiode array (PDA) detector (Shimadzu, Kyoto, Japan). The mobile phase for the LC system comprised 45% (*v*/*v*) acetonitrile, 55% (*v*/*v*) water, and 0.1% (*v*/*v*) acetic acid (isocratic conditions), and with a flow rate of 0.2 mL/min. The column temperature was set at 40 °C, and the sample injection volume was 20 μL. The wavelength of the PDA detector was set in the range of 250–330 mm.

The dissimilatory iron reduction activity of this consortium supplemented with 1 g of Mix samples and AS samples was also examined, together with blank (only medium), humin (40 g/L), rice straw only (15 g/L), and abiotic (no inoculation) controls after incubating for 7 days under the same condition described above. Amorphous Fe (III)OOH instead of sodium PCP was injected into the medium to make final concentration of 4 mM, and Fe(II) ions released by the reducing reaction were analyzed using the phenanthroline method with a spectrophotometer (U-1900, Hitachi Ltd., Tokyo, Japan) at an absorbance of 510 nm, as described in a previous study [14].

### 2.3. Chemical and Electrochemical Characterization

The elemental composition (carbon, hydrogen, nitrogen, and ash content) was analyzed using a Yanaco MT-5 CHN-corder (Yanaco New Science Inc., Kyoto, Japan), with antipyrine as the standard. Ash content was measured as the weight remaining after incineration. The oxygen content was determined by subtracting the percentages of carbon, hydrogen, nitrogen, and ash from 100%. The analysis was performed in two or three replicates. The sulfur contents of Mix-0M-C and Mix-6M-C were determined using a PerkinElmer 2400 Series II CHNS/O analyzer (PerkinElmer Japan Co., Ltd., Yokohama, Japan).

The pH was measured using an MM-60R pH meter (DKK TOA Co., Tokyo, Japan) by suspending 1 g of the sample in distilled water (2.5 g). Electrical conductivity (EC) was measured using a LAQUAtwin-EC-33B meter (HORIBA, Ltd., Kyoto, Japan) using the filtered supernatant of the suspension after the pH measurement.

Fourier transform infrared (FT-IR) spectra were measured in the range of 4000–500 cm^−1^ with a resolution of 4 cm^−1^ using a JASCO FT-IR-6100 spectrometer (JASCO, Tokyo, Japan) and the KBr method. A pure KBr pellet was used for background correction.

Electron spin resonance (ESR) spectra were acquired using a JES-FA200 ESR spectrometer (JEOL Co., Ltd., Tokyo, Japan) at 25 °C. The operation conditions were according to Pham and Katayama (2018) [56]. The first and sixth manganese (Mn) peaks were used to calibrate the g value. The effect of pH on ESR signal intensity was examined by treating 1 g of the sample with 20 mL of 0.1 M HCl or two drops of 0.1 M NaOH before freeze-drying.

Solid-state ^13^C CP/MAS nuclear magnetic resonance (NMR) spectra were obtained using an ECA-700 spectrometer (JEOL Co., Ltd., Tokyo, Japan). The operation conditions were as described by Pham and Katayama (2018) [56], with 10,000 scans for rice straw and 80,000 scans for Mix-6M-C and Mix-1Y-C. For NMR spectra, the relative abundance of different carbon groups was expressed as a percentage of the corresponding area (by integrating signal intensity with chemical shift range) of the total area (0–210 ppm) [59]. The quantitative change in each carbon group after humification was calculated by multiplying the relative abundance of each carbon group with the remaining relative carbon content of the Mix sample (assuming 100% carbon in Mix-0M-C and no change in ash content) (Calculation S2).

Cyclic voltammetry, chronoamperometry, and electrochemical impedance spectroscopy (EIS) measurements were performed using a multiple electrochemical measurement system HZ-Pro S4 (Hokuto Denko Co., Tokyo, Japan) with an electric cell, where 10 mg of the powder samples were set with a 1 mm thickness between two platinum disks (11.3 mm in diameter and 1 mm in thickness) as the working and counter electrodes, respectively, with an Ag/AgCl (3 M KCl) reference electrode (RE-T21A, EC Frontier Co., Ltd., Kyoto, Japan). The sample and electrode spaces in the electric cell were filled with N_2_-flushed 0.02 M Na_2_SO_4_ as the electrolyte. Cyclic voltammetry measurement was carried out with a scan rate of 10 mV/s and potential ranging from −0.7 to 0.4 V (vs. Ag/AgCl) for 10 cycles under anoxic conditions. The 10th cyclic voltammogram (CV) was selected to avoid oxygen contamination. Specific electric capacitance of Mix samples was estimated as shown in Calculation S3. The chronoamperometry was performed to evaluate electron accepting capacity (EAC)/electron donating capacity (EDC) of Mix samples. The sample was completely reduced at a potential of −0.6 V (vs. Ag/AgCl) for 200 min and subsequently re-oxidized at a potential of +0.5 V (vs. Ag/AgCl) for 200 min for three cycles continuously. The EDC of Mix sample was calculated as the average value of three cycles, whereas the EAC of Mix sample was calculated as the average value of the second and third cycles due to trace oxygen contamination in the first cycle (Calculation S4). EIS analysis was performed by setting the electrical frequency from 100 kHz to 1 mHz with an amplitude of 10 mV. Zview software (Schibner Associates, Inc., Southern Pines, NC, USA, version 3.5f) was employed to estimate the parameter values in the equivalent circuit in the EIS data fitting.

Raman analysis was performed using an inVia Reflex Renishaw Raman microscope (Renishaw plc., Wotton-under-Edge, Gloucestershire, UK) for Mix-0M-C and Mix-1Y-C. The measurement conditions were as follows: laser diode, 532 nm; laser power, 150 mW; exposure time of 1 s, and 10 accumulations. The Raman shift ranged from 1200 to 1800 cm^−1^. A silicon wafer was used to calibrate the Raman spectrometer.

Sulfur K-edge X-ray absorption near-edge structure spectroscopy (XANES) analysis was conducted at BL6N1 (0.85–6 keV) of the Aichi Synchrotron Radiation Center (Aichi Prefecture, Japan) for Mix-0M-C and Mix-6M-C with one sweep. The analytical procedure was performed as described by Pham et al. (2022) [58].

### 2.4. Statistical Analysis

The principal component analysis (PCA) was performed using IBM SPSS Statistics (version 21, IBM Corp., Armonk, NY, USA).

## 3. Results

### 3.1. Degradation of Rice Straw in Artificial Soil over One Year of Humification

Table 1 shows the changes in the elemental composition, pH, and EC of the Mix samples during the humification for one year. The carbon content decreased by approximately 60% over one year (Figure S2), with a decrease in hydrogen and oxygen content, assuming no change in ash content. The nitrogen content increased slightly but significantly, suggesting nitrogen fixation (Figure S3). The C/N ratio significantly decreased from 94 to 21 over one year, whereas the H/C and O/C ratios gradually increased. The pH value increased from 6.24 to 8.58 during the first month and then remained constant. The EC gradually decreased until six months and then increased.

### 3.2. Changes in EEM Functionality during the Humification of Rice Straw

**Figure 1:**
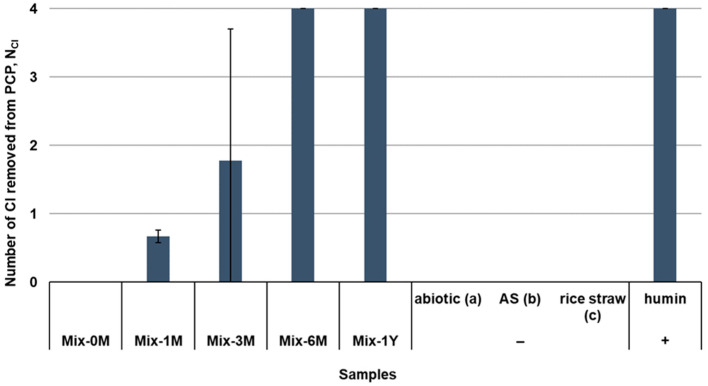
Changes in the EEM functionality of the rice straw-artificial soil mixtures with 0, 1, 3, 6 months, or 1 year of humification (Mix-0M, Mix-1M, Mix-3M, Mix-6M, and Mix-1Y), as shown by N_Cl_. The value indicates the mole numbers of Cl removed from one mole of PCP, calculated based on the proportion of PCP and its dechlorination metabolites detected in the EEM material-dependent PCP-dechlorinating cultures (third generation) (Calculation S1). Phenol was not included as a metabolite for the calculation. Positive control with humin as EEM material is shown by the symbol (+). Negative controls are indicated by the symbol (−). Negative control-a shows the representative result of abiotic controls with the Mix samples with different humification periods (Figure S6), negative control-b shows the representative result of the AS samples with different humification periods (Figure S5), and negative control-c shows no EEM functionality of rice straw itself (no artificial soil). ND denotes not detected.

The EEM functionality of the Mix samples with different humification periods was examined by the dechlorination activity as an index using the EEM material-dependent PCP dechlorinating anaerobic consortium, as shown by the number of Cl removed from PCP (Figure 1). The proportions of PCP and its dechlorination metabolites of individual samples are shown in Figure S4. The dechlorination activity was supported by humin as an EEM material (positive control) but not by the AS samples (negative control-b) (Figure 1 and Figure S5). The possibility of chemical reactions between the Mix samples and PCP was discarded because no dechlorination occurred under abiotic conditions (negative control-a) (Figure S6). Phenol was excluded in the determination of EEM functionality, as it was not only a metabolite of PCP, but degradation of rice straw itself also produced phenol as a metabolite. The phenol amount in the dechlorination culture with the Mix samples is shown in Figure S7.

**Figure 2:**
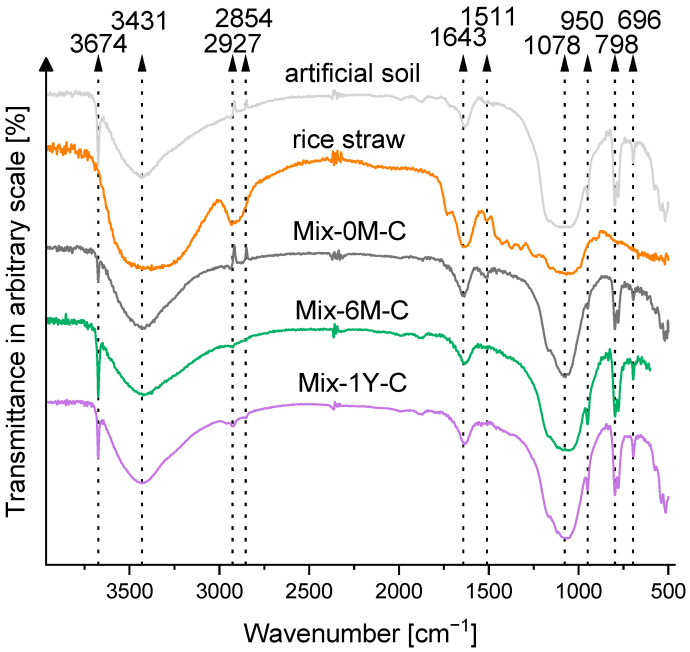
FT-IR spectra of rice straw-artificial soil mixtures with 0 and 6 months, and 1 year of humification (Mix-0M-C, Mix-6M-C, Mix-1Y-C), rice straw only, and artificial soil only.

**Figure 3:**
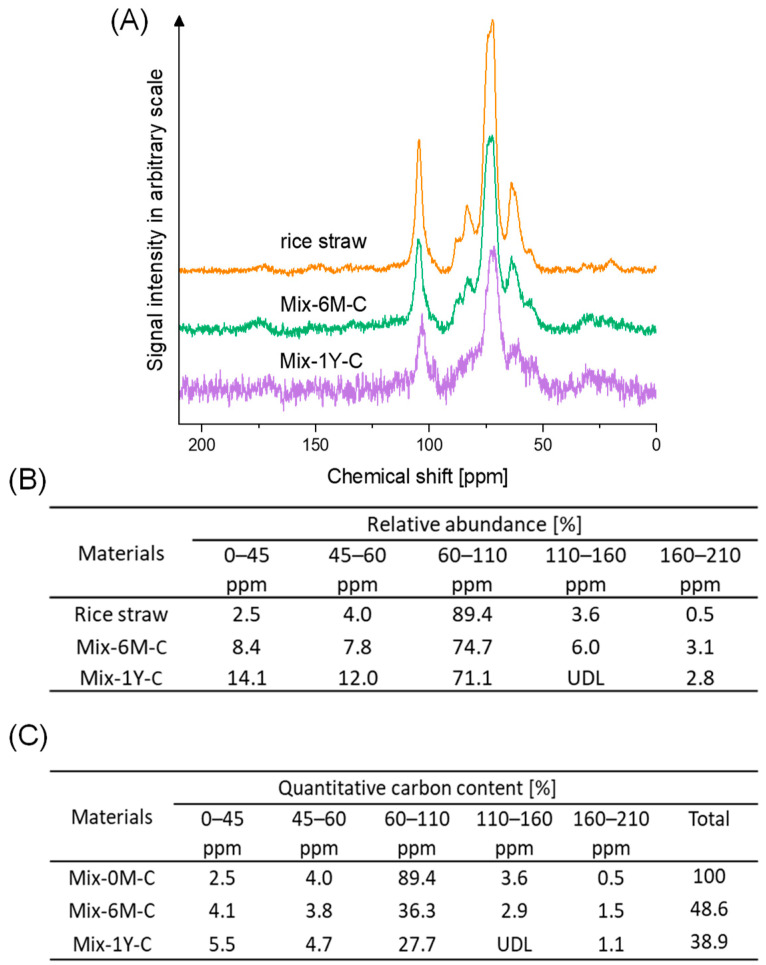
(**A**): ^13^C CP/MAS NMR spectra of rice straw-artificial soil mixtures with six months and one year of humification (Mix-6M-C, Mix-1Y-C) and rice straw only. (**B**): The relative abundance of different carbon groups in ^13^C CP/MAS NMR spectra calculated by integrating signal intensities with chemical shift range and expressed as percentages of the total area (0–210 ppm). (**C**): Quantitative changes in carbon groups based on elemental analysis and ^13^C CP/MAS NMR spectra. Assignment of different carbon groups was as follows: 0–45 ppm, alkyl carbon; 45–60 ppm, N-alkyl carbon; 60–110 ppm, carbohydrate carbon; 110–160 ppm, aromatic carbon; and 160–210 ppm, carbonyl carbon [59]. The UDL was below the detection limit, where the signal-to-noise ratio was smaller than 2.

**Figure 4:**
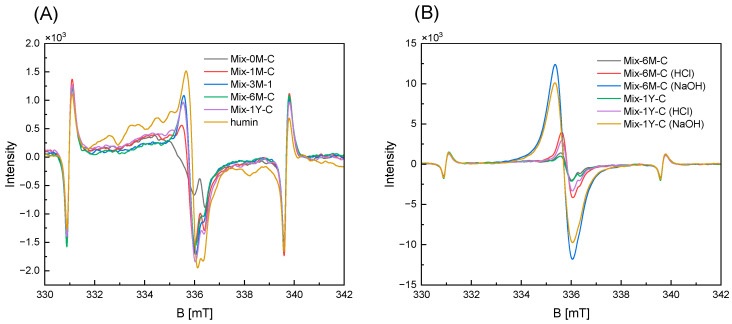
(**A**): The ESR spectra of rice straw-artificial soil mixtures with 0, 1, 3, 6 months, or 1 year of humification (Mix-0M-C, Mix-1M-C, Mix-3M-1, Mix-6M-C, and Mix-1Y-C), and of humin, with the 3rd and 4th Mn marker signals. The measurement was carried out using the composite samples except for Mix-3M-1. (**B**): The ESR spectra of rice straw-artificial soil mixtures with 6 months and 1 year of humification (Mix-6M-C, Mix-1Y-C) and Mix-6M-C and Mix-1Y-C treated with 0.1 M HCl or 0.1 M NaOH, respectively, with the 3rd and 4th Mn marker signals. The measurement was carried out using the composite samples.

Based on the dechlorination activity, it was demonstrated for the first time that EEM functionality emerged during the humification of rice straw in artificial soil. PCP dechlorination was not observed in rice straw alone (negative control-c) or Mix-0M, indicating that rice straw had no EEM functionality. However, after one month of humification, Mix-1M showed activity as an EEM material, where approximately 0.7 number of Cl was removed from PCP (Figure 1) and 2,3,4,5-TeCP and 3,4,5-trichlorophenol (3,4,5-TCP) were detected as the dechlorination metabolites of PCP (Figure S4). The EEM functionality increased with time, as shown by the increase in number of Cl removed from PCP of Mix samples. After three months of humification, one Mix-3M sample showed stronger EEM functionality (Figure S4), shown as the wide deviation Mix-3M. After six months, all Mix-6M samples exhibited strong EEM functionality, comparable to that of the positive control, humin. This strong EEM functionality of the Mix samples was maintained after one year of humification. The emergence of EEM functionality was replicative in the second humification experiment of Mix (Mix2) (Figure S8).

Examination of dissimilatory iron reduction activity of the samples was also performed (Figure S9), as it is widely used to examine the EEM functionality. The Mix samples exhibited stronger dissimilatory iron reduction activity than those of abiotic controls and AS samples, as more Fe(II) ions were reduced when supplemented with the Mix samples. However, rice straw only and all Mix-0M samples showed the highest iron reduction activity (Figure S9A,D). The results suggested that rice straw itself could be utilized as carbon sources/electron donors in this microbial consortium for dissimilatory iron reduction. Therefore, the PCP dechlorination activity in an anaerobic EEM material-dependent PCP-dechlorinating consortium was used as an index to determine the EEM functionality of materials rather than dissimilatory iron reduction activity, which enabled us to distinguish the role of materials as EEM materials and effectively examine the EEM functionality of Mix samples.

### 3.3. Changes in Chemical Structures during the Humification

Figure 2 shows the representative FT-IR spectra of Mix-0M-C to Mix-1Y-C. The FT-IR spectra of individual samples are shown in Figure S10. Small peak at 1511 cm^−1^, assigned to aromatic skeletal stretching in the lignin of rice straw [60], disappeared with the humification process. Other peaks did not show evident changes during humification: the peaks at 2927 and 2854 cm^−1^ assigned to C-H stretching of aliphatic groups, a sharp peak at 3674 cm^−1^ assigned to O-H stretching [61], the broad peak at 3431 cm^−1^ assigned to O-H stretching from both artificial soil [62] and rice straw, and N-H stretching from rice straw, the peak at 1643 cm^−1^ assigned to the physical adsorption of water O-H vibration in kaolin [63] and C=O stretching in lignin/hemicellulose of rice straw [64], the strong peak at 1078 cm^−1^ assigned to Si-O stretching of artificial soil and C-O stretching of cellulose and hemicellulose in rice straw, and the peaks at 950, 798, and 696 cm^−1^ assigned to Si-O and Al-OH of artificial soil [65,66].

Figure 3A shows ^13^C CP/MAS NMR spectra of Mix-6M-C and Mix-1Y-C compared with that of rice straw. The changes in the contents of different carbon groups were estimated from the NMR spectra and the total carbon content, as summarized in Figure 3B,C, respectively (Calculation S2). The alkyl carbon (0–45 ppm) increased from 2.5% in Mix-0M-C to 5.5% in Mix-1Y-C, in agreement with the increase in the H/C ratio in the elemental analysis. The slight increases in N-alkyl (45–60 ppm) and carbonyl (160–210 ppm) carbons were consistent with the increases in nitrogen content and O/C ratio in the elemental analysis. Carbohydrate carbon (60–110 ppm) was always dominant in the Mix samples, although it dramatically decreased from 89.4% to 27.7% during humification, indicating the decomposition of cellulose and hemicellulose in rice straw. The carbons observed at 114–117, 127–140, and 140–154 ppm in rice straw could be assigned to the lignin fraction [67]. The aromatic carbon (110–160 ppm) in rice straw and Mix-6M-C disappeared in Mix-1Y-C.

ESR spectra showed an increase in organic radicals in the Mix samples during humification (Figure 4A). Mix-0M-C did not exhibit the signal of rice straw (g = 2.0040, Figure S11) but mainly exhibited the signal of artificial soil in the spectrum. The signal intensity increased in Mix-3M-1 and remained stable in the Mix samples over one year, with g-values ranging from 2.0035 to 2.0043, which were assigned to organic radicals. Despite the difference in EEM functionality among the three replicates of Mix-3M, there was no considerable difference in signal intensity among the three replicates (Figure S12). The signal intensities of Mix-6M-C and Mix-1Y-C significantly increased when treated with HCl/NaOH solution, especially under alkaline conditions (Figure 4B). The increase in organic radicals in the Mix2 samples was reproduced in the second experiment (Figure S13).

### 3.4. Changes in Electrochemical Properties during the Humification

**Figure 5:**
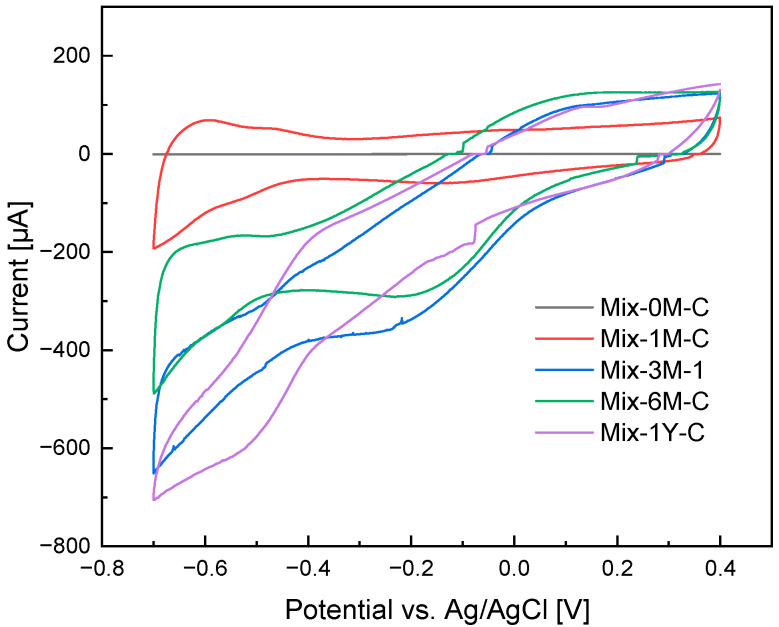
Cyclic voltammograms (CVs) of rice straw-artificial soil mixtures with 0, 1, 3, 6 months, and 1 year of humification (Mix-0M-C, Mix-1M-C, Mix-3M-1, Mix-6M-C, and Mix-1Y-C). All CVs presented here were obtained at the 10th cycle of the measurement. The measurement was carried out using the composite samples except for Mix-3M-1.

The CVs demonstrated that the Mix sample was not initially redox-active, but converted to redox-active during humification (Figure 5). Currents of less than 3 μA were detected in the CVs of rice straw and Mix-0M-C (Figure S14). After three months of humification, the CVs showed larger currents to the applied voltage and overall slope, especially when applied negative potential. However, no specific redox peaks were identified for any of the Mix samples. This suggested an increase in the redox-active moieties in the samples.

**Figure 6:**
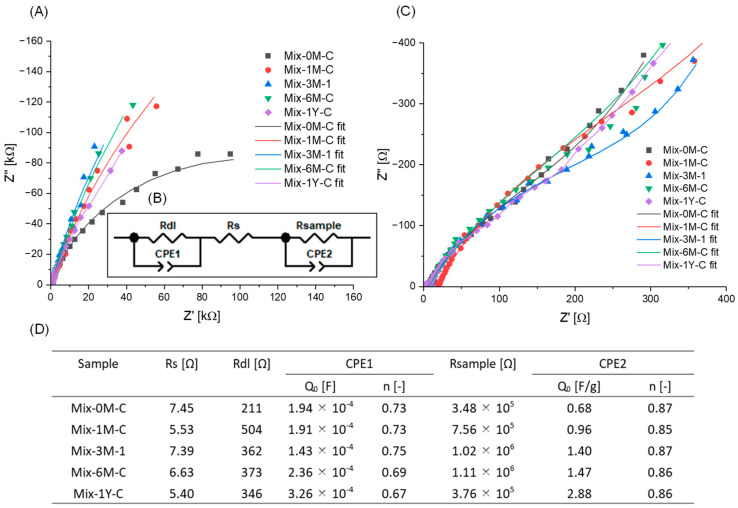
(**A**) Electrochemical impedance spectra (EIS) (Nyquist plots) and fitting curves of Mix-0M-C, Mix-1M-C, Mix-3M-1, Mix-6M-C, Mix-1Y-C. (**B**) Equivalent circuit model used for fitting the EIS data. Rdl: resistance of electrode double layer; CPE1: constant phase element for electrode double layer; Rs: resistance of the solution; Rsample: resistance of the sample; CPE2: constant phase element for sample; Q_0_ and n are frequency-independent parameters of CPE. (**C**) Nyquist plots and fitting curves on high-frequency range. (**D**) Table of fitting parameters.

EIS measurements were performed under the same conditions as the cyclic voltammetry measurements to obtain the electric capacitance of Mix samples. Using the EIS Nyquist plots and curve fittings based on the equivalent circuit, the electric parameters of the Mix samples were estimated (Figure 6). Curve fittings were also confirmed in Bode plots (Figure S15). Two constant phase elements (CPE) were introduced for the electrode double layer (CPE1) and the sample surface (CPE2) due to the porous property of the samples, as previously suggested [68,69]. The Q_0_ value of CPE2, as analogous to capacitance, was used to estimate the electric capacitance of the Mix samples since they had n values larger than 0.8 [70]. The specific electric capacitance per unit gram carbon of the Mix samples, CPE2-Q_0_/gC, increased from 0.82 to 2.88 F/gC during humification.

**Figure 7:**
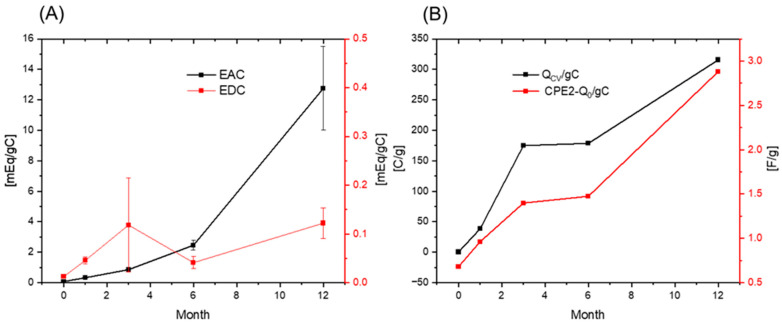
(**A**) Electron accepting capacity (EAC) and electron donating capacity (EDC) of rice straw-artificial soil mixtures with 0, 1, 3, 6 months, and one year of humification (Mix-0M-C, Mix-1M-C, Mix-3M-1, Mix-6M-C, and Mix-1Y-C). (**B**) Changes in the specific electric capacitance per carbon based on CV measurement, Q_CV_/gC (Calculation method is shown in Supplementary Information Calculation S3) and the electric capacitance per carbon estimated by EIS, CPE2-Q_0_/gC, in the Mix samples during humification.

The specific electric capacitance per unit gram of carbon in the Mix samples based on CV measurement, Q_CV_/gC (Calculation S3), increased from Mix-0M-C to Mix-1Y-C, agreeing with an increase in the specific electric capacitance of the Mix samples over one year of humification in EIS analysis, CPE2-Q_0_/gC (Figure 7B). In addition to CV and EIS analysis, the chronoamperometry of Mix samples was also conducted to evaluate their EAC/EDC, as it was widely used as an index for electron capacity of the material. The EAC (1.65–14.69 mEq/gC) of Mix samples was much higher than their EDC (0.17–0.64 mEq/gC). The EAC of Mix sample slightly decreased during the first-month of incubation, and then increased to 14.69 mEq/gC until humification for one year, whereas their EDC decreased to 0.17 mEq/gC after three months and then increased to 0.64 mEq/gC for one-year humification, as shown in Figure 7A (Calculation S4). The results suggested that the increase in electric capacitance of Mix samples during one-year humification mainly contributed to the increase in their EAC.

## 4. Discussion

This study demonstrated the emergence of EEM functionality in a rice straw-artificial soil mixture during humification for over one year for the first time (Figure 1). The EEM functionality of the freeze-dried samples was evaluated using an EEM material-dependent PCP dechlorinating anaerobic consortium. Traditional humic substances studies have been performed for samples extracted using strong acid, alkaline, or organic solvents, which results in “selective preservation” of a specific structure and alteration of its original chemical structure [3,71]. In this study, the EEM functionality was examined using freeze-dried samples. Therefore, the emergence of functionality was considered to reflect the changes in the Mix samples during humification, without any alteration. Rice straw is not an EEM material. However, after one month of humification in artificial soil, the Mix-1M samples showed EEM functionality, as shown by dechlorination activity. The EEM functionality of the Mix samples increased until six months and was maintained until one year. It should be noted that the inoculated AS samples did not show any EEM functionality during one year of humification (Figure S5), indicating that the emergence of EEM functionality resulted from the humification of rice straw rather than the weathering of artificial soil. However, Wang and Huang (1989) reported that kaolinite and quartz had catalytic power and contained reactive sites for the formation of hydroquinone-derived polymers from the mixture of phenols [72], which may catalyze the humification of rice straw and support the emergence of EEM functionality in rice straw-artificial soil mixture. When the EEM material-dependent PCP dechlorinating consortium was incubated in organic carbon source-free medium with the Mix samples, PCP was still dechlorinated in cultures with Mix-1M, 3M, 6M, and 1Y samples (Figure S16). The results indicate that the Mix samples played a dual role as electron donors and redox mediators in PCP dechlorination.

Rice straw comprised three main components: cellulose, hemicellulose, and lignin, which were observed in FT-IR (Figure 2 and Figure S10) and NMR spectra (Figure 3). The carbon content of rice straw rapidly degraded more than 22% (Figure S2) in the first month of incubation, which would be mainly caused by the degradation of cellulose and hemicellulose, as they were easily attacked by cellulolytic organisms and decompose more rapidly than aromatic polymer lignin [51]. Although the aromatic skeletal stretching of lignin was still observed in the FT-IR spectra after one-month humification of rice straw-artificial soil mixture (Mix-1M-C) (Figure S10), the increase in organic radicals was already observed in the ESR spectra (Figure 4). This would be due to the partial degradation of lignin to phenol monomer, which acted as the precursor for the synthesis of quinone structure via polyphenol theory, as suggested by a previous study [73]. This increase in organic radical (ESR spectra in Figure 4) and carbonyl carbon (NMR spectra in Figure 3) suggested that quinone structures were progressively formed until six months, accompanied by the further degradation of cellulose, hemicellulose, and lignin structures in rice straw, indicated by CHN contents (Table 1), FT-IR spectra (Figure 2 and Figure S10), and NMR spectra (Figure 3). From six months to one year, rice straw was further decomposed but slowly (CHN contents in Table 1 and NMR spectra in Figure 3), with a slight increase in aliphatic and N-alkyl carbons (NMR analysis in Figure 3), whereas quinone structures were maintained (ESR spectra in Figure 4).

**Figure 8:**
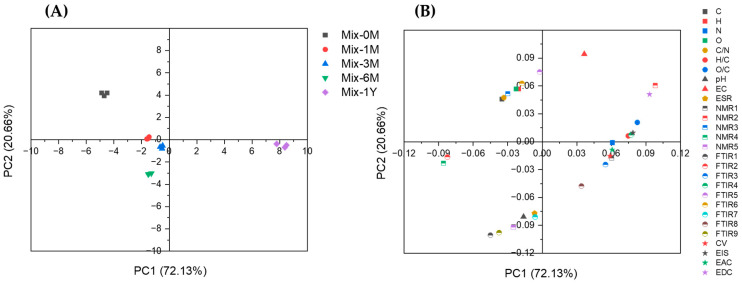
Principle component analysis of rice straw-artificial soil mixtures (Mix) with different humification periods (**A**) and loadings of various chemical and electrochemical parameters (**B**). C, H, N, and O denote carbon, hydrogen, nitrogen, and oxygen percentage of Mix sample, respectively (assuming no change in ash content); C/N, H/C, and O/C denote carbon to nitrogen, hydrogen to carbon ratio, and oxygen to carbon ratio of Mix sample, respectively (elemental ratio); pH denotes pH value of Mix sample; EC denotes electrical conductivity of Mix sample; ESR denotes signal intensity of Mix sample in ESR analysis; NMR1, 2, 3, 4, and 5 denote quantitative carbon content of alkyl carbon (0–45 ppm), N-alkyl carbon (45–60 ppm), carbohydrate carbon (60–110 ppm), aromatic carbon (110–160 ppm), and carbonyl carbon (160–210 ppm), respectively (Figure 3C); FTIR1, 2, 3, 4, 5, 6, 7, 8, and 9 denote relative intensity of O-H (3674 cm^−1^), O-H and N-H (3431 cm^−1^), C-H (2927 cm^−1^), C-H (2854 cm^−1^), O-H of kaolin and C=O of lignin/hemicellulose (1643 cm^−1^), aromatic skeleton of lignin (1511 cm^−1^), Si-O in artificial soil (950 cm^−1^), Al-OH of artificial soil (798 cm^−1^), and Si-O in artificial soil (696 cm^−1^) in the FT-IR spectra, respectively (with C-O/Si-O (1078 cm^−1^) as the base); CV denotes Q_CV_/gC (Calculation S3); EIS denotes CPE2-Q_0_/gC (Figure 6); EAC denotes electron accepting capacity of Mix sample; EDC denotes electron donating capacity of Mix sample (Figure 7A).

The changes in the chemical and electrochemical properties of the Mix samples were compared with the increase in EEM functionality with reference to PCA (Figure 8). For six months, with an increase in EEM functionality, the degradation of organic matter in rice straw was conspicuous, as indicated by the decrease in carbon, hydrogen, and oxygen. This correlation was also observed for the Mix-3M samples. Mix-3M-3 showed stronger EEM functionality than Mix-3M-1 and Mix-3M-2, consistent with the higher degree of rice straw degradation (Table S2) and higher phenol content in Mix-3M-3. There was also a decrease in the peak intensity of the aromatic skeleton in lignin (FTIR6 in Figure 8) and carbohydrate carbon (NMR3 in Figure 8), indicating the degradation of cellulose, hemicellulose, and lignin fractions in rice straw. However, increases in the signal intensity of organic radicals (ESR) and carbonyl carbon (NMR5 in Figure 8) were observed, accompanied by an increase in pH and a decrease in EC. An increased response of the signal intensities in ESR to alkaline than to acid was also observed (Figure 4B), a typical response for semi-quinone-type radicals in humic substances [8,10]. This indicated the synthesis of the quinone structure during the six months of humification, although the presence of other organic radicals produced during humification should also be considered. An increase in the specific electric capacitance was also observed. These results suggest that the synthesis of quinone structures increases the EEM functionality and the specific electric capacitance during the decomposition of the cellulose/hemicellulose and lignin fractions in the Mix samples. During the period from six months to one year of humification, when the EEM functionality was maintained, increases in nitrogen content (Table 1 and Figure S3), O/C ratio, and N-alkyl carbon (NMR2 in Figure 8) were observed, indicating nitrogen fixation and further oxygenation of the Mix samples. The increases in alkyl carbon (NMR1 in Figure 8), C-H peak intensity (FT-IR3 and 4 in Figure 8), and H/C ratio also indicated an increase in aliphatic structures, whereas aromatic carbon (NMR4 in Figure 8) decreased. These changes may contribute to the maintenance of EEM functionality despite the decrease in organic matter content from six months to one year. This was supported by the increase in the electric capacitance/capacity per unit gram of carbon (indicated by CV, EIS, EAC, and EDC) of the Mix samples during this period (Figure 7).

Although the phenolic compounds can be polymerized to polyphenol or quinone [4,32,74] and converted into redox-active centers in the Mix samples, phenol itself was not considered responsible for the EEM functionality, as it was detected even in the non-functional Mix-0M samples (Figure S7). ESR analysis suggested that quinone structures were newly synthesized in the Mix samples during humification. The increase of quinone structures, accompanied by the increase in electron transfer capacity (EAC + EDC) of humic acid was also observed during corn straw composting process recently [73]. They suggested that lignin in corn straw decomposed into phenol monomer, which acted as precursor for the synthesis of quinone structure in humic acid of humified corn straw compost via polyphenol theory [73].

The selected freeze-dried microbial biomass did not enable the dechlorination activity, that is no EEM functionality (Figure S17). This supports the hypothesis that the newly synthesized redox-active structures in the Mix samples during humification are responsible for the EEM functionality. Although sulfur and sulfur-containing functional groups have been reported as potential redox-active centers in humin [58], the sulfur contents of the Mix samples were below the detection limit (Table 1). Therefore, sulfur and sulfur-containing functional groups are unlikely to be the main redox-active centers in the Mix samples. However, the oxidation states of sulfur in the Mix sample could change, as shown by sulfur K-edge XANES (Figure S18). Although the β-sheet secondary structure was found to be the main component of EEM functionality in silk protein [75], and proteins have been reported to be preserved in the structure of humic substances [76], this was not the case for the Mix samples because the β-sheet structure was not detected in the Raman spectra (Figure S19).

## 5. Conclusions

This study demonstrated for the first time that EEM functionality emerged during the humification of rice straw in artificial soil, and its functionality was maintained for one year. The emergence of EEM functionality was correlated with the degradation of rice straw, the formation of quinone structure and the increase in specific electric capacitance. The newly formed quinone structure was suggested as the potential redox-active center for the EEM functionality, and nitrogenous and aliphatic structures may be associated with the maintenance of functionality. The humification of rice straw suggested that the EEM functionality in humic substances would not be originally present in the fresh organic material, but emerged by humification. In the second humification experiment, the Mix2 samples showed the emergence of EEM functionality again, but the functionality decreased after one year (Figure S6). This was probably due to the faster degradation of rice straw accompanied by the earlier emergence of EEM functionality in the second experiment, where the easily utilized carbon source in rice straw may be used up in the incubation bottle so that the newly formed quinone was used as the carbon source for microbes and further degraded. Thus, the EEM functionality emerges first during the degradation of organic matter, is maintained for some time, and may finally disappear after further degradation of organic matter. Further long-term studies are required to evaluate whether the EEM functionality is preserved during long-term humification. However, this study demonstrated a one-year change in EEM functionality during the humification process of rice straw, a natural lignocellulosic material. This opens a new view of dynamic changes in EEM functionality. In addition, because humic substances are formed from various origins, the humification processes of other types of organic materials, such as proteinaceous and lipid materials, are worth studying in the future.

## Figures and Tables

**Table 1:** Elemental composition, pH, and EC of rice straw-artificial soil mixtures.

Incubation Time	Elemental Composition [% w/w]				Ash [% w/w]	Elemental Ratio			pH	EC [μS/cm]
	C	H	N	O		C/N	H/C	O/C		
0 month	4.03 (0.28)	0.71 (0.04)	0.05 (0.01)	5.62 (0.47)	89.60 (0.80)	94.4 (7.2)	2.10 (0.02)	1.04 (0.01)	6.24 (0.03)	1766 (63)
1 month	3.14 (0.13)	0.61 (0.02)	0.07 (0.01)	4.43 (0.18)	91.75 (0.34)	52.5 (1.8)	2.32 (0.01)	1.06 (0.00)	8.58 (0.12)	1516 (40)
3 months	2.68 (0.18)	0.53 (0.04)	0.08 (0.00)	3.93 (0.32)	92.79 (0.54)	39.0 (2.7)	2.36 (0.03)	1.10 (0.02)	8.82 (0.31)	1432 (10)
6 months	2.06 (0.03)	0.44 (0.01)	0.07 (0.00)	3.13 (0.10)	94.31 (0.14)	36.2 (1.7)	2.54 (0.03)	1.14 (0.02)	8.84 (0.10)	1377 (23)
One year	1.66 (0.23)	0.44 (0.05)	0.09 (0.01)	3.03 (0.29)	94.78 (0.58)	21.1 (2.4)	3.18 (0.27)	1.38 (0.06)	8.23 (0.16)	1562 (43)

Note. Numbers in parenthesis show the standard deviation. Oxygen content was obtained by subtraction of other elements and ash. The sulfur content was below the detection limit (0.3%).

## Data Availability

The data presented in this study are available in the Supplementary Materials.

## Supplementary Materials

The following supporting information can be downloaded at: https://www.mdpi.com/article/10.3390/ijerph192215173/s1, Table S1: elemental composition of rice straw; Table S2: elemental composition of Mix-3M; Calculation S1: estimation of number of Cl removed from PCP, N_Cl_; Calculation S2: quantitative changes in carbon groups; Calculation S3: estimation of specific electric capacitance by CV analysis; Calculation S4: estimation of EAC/EDC; Figure S1: proportions of PCP and its metabolites in the humin cultures for different concentrations; Figure S2: degradation of rice straw during the humification; Figure S3: statistical analysis of N contents for Mix samples; Figure S4: proportions of PCP and its metabolites in the cultures for individual Mix sample; Figure S5: no EEM functionality in abiotic controls of Mix samples (negative control-a); Figure S6: no EEM functionality AS samples (negative control-b); Figure S7: changes in phenol amount in the cultures with the Mix samples; Figure S8: changes in EEM functionality of Mix2 samples in the second time experiment; Figure S9: dissimilatory iron reduction activity; Figure S10: FT-IR spectra of individual Mix sample; Figure S11: ESR spectra of kaolin, sand, and rice straw; Figure S12: ESR spectra of Mix-3M; Figure S13: ESR spectra of Mix2 samples in the second time experiment; Figure S14: cyclic voltammograms of rice straw and Mix-0M-C; Figure S15: EIS Bode plots of Mix samples; Figure S16: changes in EEM functionality of Mix samples in the culture without formate; Figure S17: no EEM functionality of selected microbial biomass; Figure S18: sulfur XANES spectra of Mix-0M-C and Mix-6M-C; Figure S19: Raman spectra of Mix-0M-C and Mix-1Y-C.
