# Supplementary material for: The Emergence of Extracellular Electron Mediating Functionality in Rice Straw-Artificial Soil Mixture during Humification
Source: Int J Environ Res Public Health. 2022 Nov 17;19(22):15173. doi: 10.3390/ijerph192215173 (PMC9691237; doi:10.3390/ijerph192215173)
Supplement: Supplementary file 1 [file ijerph-19-15173-s001.zip › ijerph-1933621-supplementary.pdf]

Supplemental Information for

The Emergence of Extracellular Electron Mediating Functionality in Rice Straw-artificial Soil Mixture During Humification

Tingting Hu [1], Duyen Minh Pham [2], Takuya Kasai [1,2] and Arata Katayama [1,2,*]

[1] Graduate School of Engineering, Nagoya University, Tokai National Higher Education and Research System, Chikusa, Nagoya 464-8603, Japan

[2] Institute of Materials and Systems for Sustainability, Nagoya University, Tokai National Higher Education and Research System, Chikusa, Nagoya 464-8603, Japan

* Correspondence: katayama.arata@nagoya-u.jp; Tel.: +81-(0)52-789-5856

Number of Pages: 22

Number of Tables: 2

Number of Method (Calculation): 4

Number of Figures: 19

Table S1. Elemental composition of rice straw.

Material	Elemental composition [% w/w]				Ash	Elemental ratio		
	C	H	N	O	[% w/w]	C/N	H/C	O/C
Rice straw	39.21 ±0.02	5.78 ±0.12	0.56 ±0.02	41.46 ±0.33	12.99 ±0.44	81.24 ±2.23	1.77 ±0.04	0.79 ±0.01

* Oxygen content was obtained by subtraction of other elements and ash.

Table S2. Elemental composition of three replicates of rice straw-artificial soil mixtures with 3 months of humification (Mix-3M).

Materials	Elemental composition [% w/w]				Ash	Elemental ratio		
	C	H	N	O	[% w/w]	C/N	H/C	O/C
Mix-3M-1	2.74	0.54	0.08	4.10	92.54	39.91	2.38	1.12
Mix-3M-2	2.82	0.56	0.08	4.12	92.41	41.17	2.38	1.10
Mix-3M-3	2.47	0.48	0.08	3.56	93.41	36.02	2.33	1.08

Calculation S1. Estimation of number of chorine (Cl) removed from PCP, as index of EEM functionality of the sample, based on the proportion of PCP and its metabolites of dechlorination activity.

$$N_{Cl} = \sum_{i=1}^{5} N_{Cl_i} \times \eta_i$$

where N_{Cl} in the dimensionless number, meaning the mole number of Cl removed from one mole of PCP; N_{Cl_i} is the dimensionless number, denoting the mole number of Cl removed from one mole of PCP for each metabolite (PCP, 2,3,4,5-TeCP, 3,4,5-TCP, 3,5-dichlorophenol (3,5-DCP), and 3-CP have 0, 1, 2, 3, and 4 of N_{Cl_i}, respectively); η_i denotes the proportion of PCP and each metabolite after the incubation, expressed as mole percentage [mol/mol]; i denotes numbers 1, 2, 3, 4, and 5 indicating PCP, 2,3,4,5-TeCP, 3,4,5-TCP, 3,5-DCP, and 3-CP, respectively. Since, phenol is not counted for the calculation in this study, the maximum value of N_{Cl} is 4.

Calculation S2. Quantitative changes in carbon groups based on elemental analysis and ^{13}C CP/MAS NMR spectra.

The calculation to obtain the quantitative carbon content was performed as follows: For Mix-0M-C, the ^{13}C CP/MAS NMR spectrum of rice straw was used to evaluate the relative abundance of different carbon groups because rice straw was the sole organic matter (trace organic carbon in the soil suspension inoculum and artificial soil was negligible) in Mix-0M-C. The carbon content of Mix-0M-C was set to 100%, and the relative remaining carbon contents (Mrc) of Mix-6M-C and Mix-1Y-C were 48.6% and 38.9%, respectively, as shown in Supplemental Information Figure S2. The quantitative carbon content (Mqc) was calculated using the following equation.

$$Mqc\ [\%] = P_{Ci} \times Mrc\ [\%]$$

where P_{Ci} denotes the relative abundance of different carbon groups in Mix-6M-C/Mix-1Y-C [%] (Figure 3B).

Calculation S3. Estimation of the specific electric capacitance of rice straw-artificial soil mixtures in cyclic voltammetry analysis, indicated by Q_{cv}/gC.

$$Q_{CV} = \int_{t_1}^{t_2} (A_{forward} - A_{reverse}) \times dt \quad (1)$$

$$CV\ area = \int_{V_1}^{V_2} (A_{forward} - A_{reverse}) \times dV \ (2)$$

$$V = \nu \times t \ (3)$$

Inserts (3) into (2), thus

$$CV\ area = \int_{t_1}^{t_2} (A_{forward} - A_{reverse}) \times \nu dt = \nu \int_{t_1}^{t_2} (A_{forward} - A_{reverse}) \times dt = \nu \times Q_{CV}$$

$$Q_{CV} = \frac{CV\ area}{\nu}$$

where Q_{CV} [C] denotes the charge stored in the sample during CV measurement; V_1 [V] and V_2 [V] denote the voltage range in the cyclic voltammetry measurement; $A_{forward}$ [A] and $A_{reverse}$ [A] denote the currents observed in the forward and reverse CV, respectively; V [V] denotes the voltage; ν [V/s] denotes the scan rate of CV analysis, which is 0.01 V/s in this study; t [s] denotes time.

$$Q_{CV}/gC\ [C/g] = Q_{CV}\ [C]\ /\ Mc\ [g]$$

where Mc denotes the mass of the carbon in the Mix sample. Mc is obtained using the following equation:

$$Mc = 0.01\ [g] \times \text{carbon content in the Mix sample}$$

where 0.01 [g] is the amount of the Mix sample used for the measurement of cyclic voltammetry, and the carbon content in the Mix samples is listed in Table 1.

Calculation S4. Estimation of the electron donating capacity (EDC)/electron accepting capacity (EAC) of rice straw-artificial soil mixtures in chronoamperometry analysis.

$$\mathrm{EAC_i/EDC}_i = \frac{\left(\int_0^t \mathrm{I_t dt}\right)_i}{\mathrm{F \times M_c}}$$

$$\mathrm{EAC} = \frac{\mathrm{EAC_2 + EAC_3}}{2}$$

$$\mathrm{EDC} = \frac{\mathrm{EDC_1 + EDC_2 + EDC_3}}{3}$$

where EAC [mEq/gC] denotes electron accepting capacity; EDC [mEq/gC] denotes electron donating capacity; I_t denotes the current during chronoamperometry measurement; t denotes the measurement time (200 min); i denotes the number of cycle; F denotes Faraday constant (96485.3328 C/mol); Mc denotes the mass of the carbon in the Mix sample. Mc is obtained using the following equation:

$$\mathrm{Mc} = 0.01\ [\mathrm{g}] \times \text{carbon content in the Mix sample}$$

where 0.01 [g] is the amount of the Mix sample used for the measurement of chronoamperometry, and the carbon content in the Mix samples is listed in Table 1.

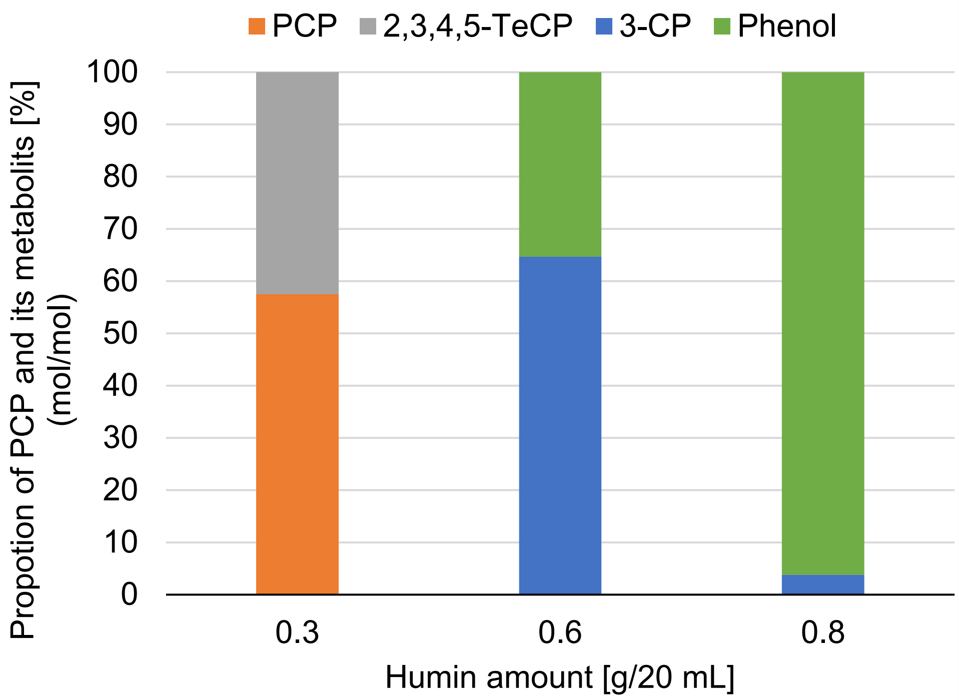

Figure S1. The proportion of PCP and its dechlorination metabolites detected in the EEM material-dependent PCP-dechlorinating cultures with different amount of humin in 20 mL medium.

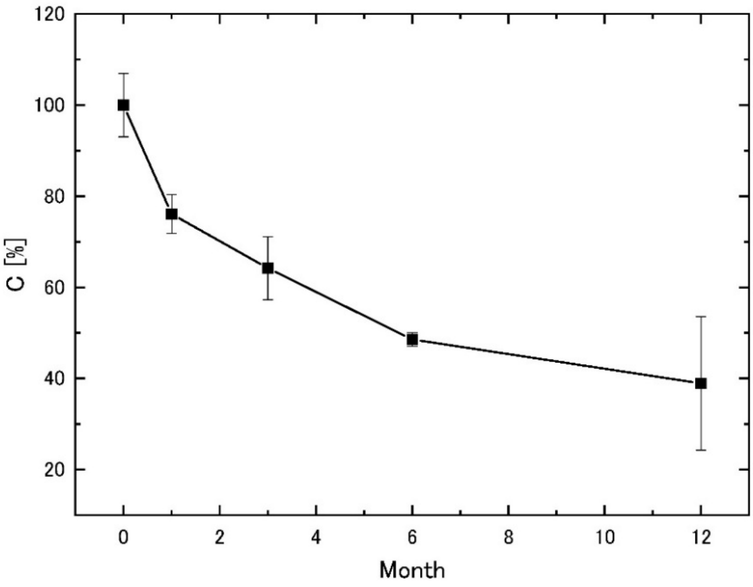

Figure S2. Degradation of rice straw during the humification process over one year as indicated by carbon content (assuming no change in the ash amount).

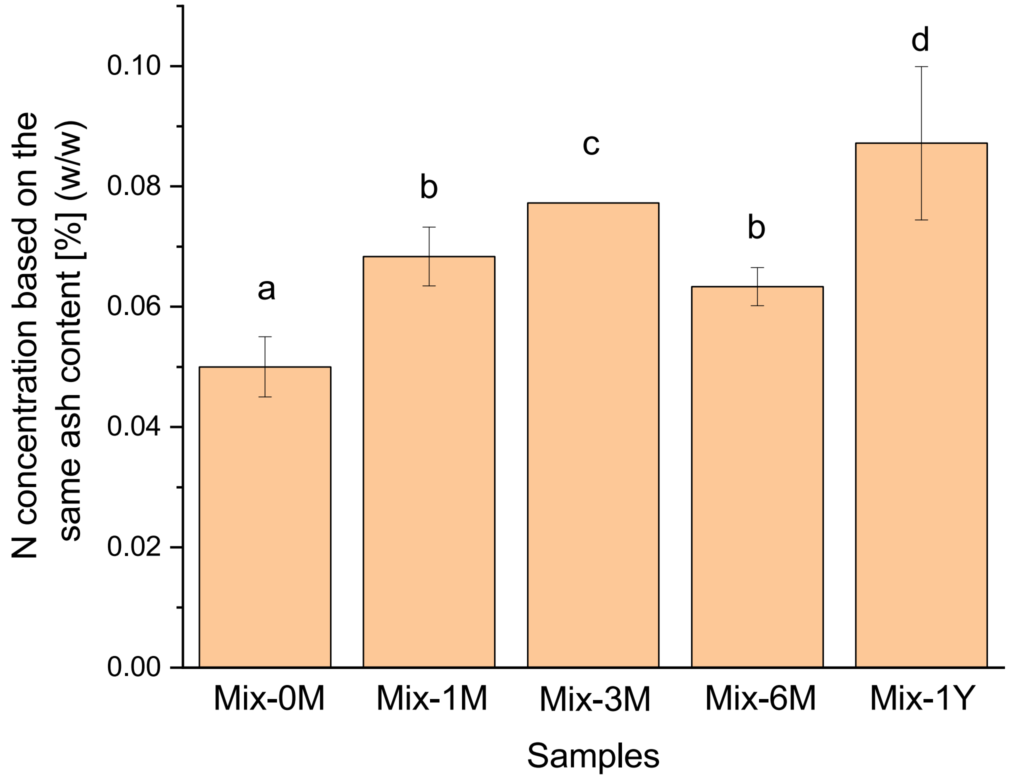

Figure S3. Nitrogen concentration (weight percentage) of rice straw-artificial soil mixtures with different humification periods, according to CHN analysis (based on the same ash content). Different letters show the statistically significant difference (p < 0.05) in one-way ANOVA followed by Tukey's post hoc test.

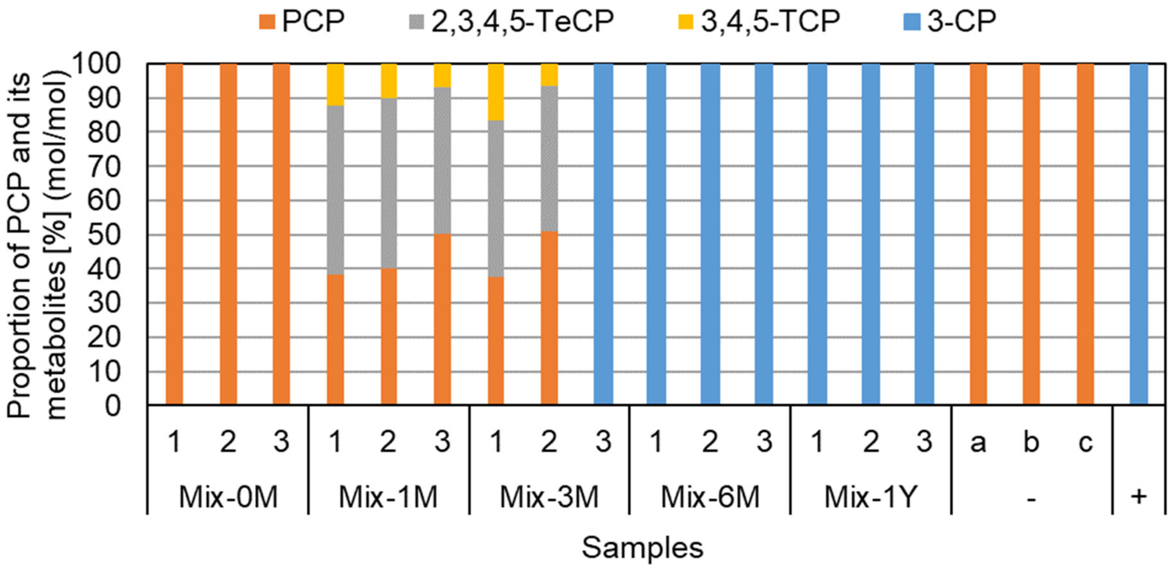

Figure S4. Changes in the EEM functionality of the rice straw-artificial soil mixtures with 0, 1, 3, 6 months, or one year of humification (Mix-0M, Mix-1M, Mix-3M, Mix-6M, and Mix-1Y), as shown by the proportion of PCP and its dechlorination metabolites detected in the EEM material-dependent PCP-dechlorinating cultures (third generation) as an index. Phenol was not included as a metabolite. Numbers 1, 2, and 3 represent triplicate samples. Positive control with humin as EEM material is shown by the symbol (+). Negative controls are indicated by the symbol (–). Negative control-a shows the representative result of abiotic controls with the Mix samples with different humification periods (Figure S6), negative control-b shows the representative result of the AS samples with different humification periods (Figure S5), and negative control-c shows no EEM functionality of rice straw itself (no artificial soil).

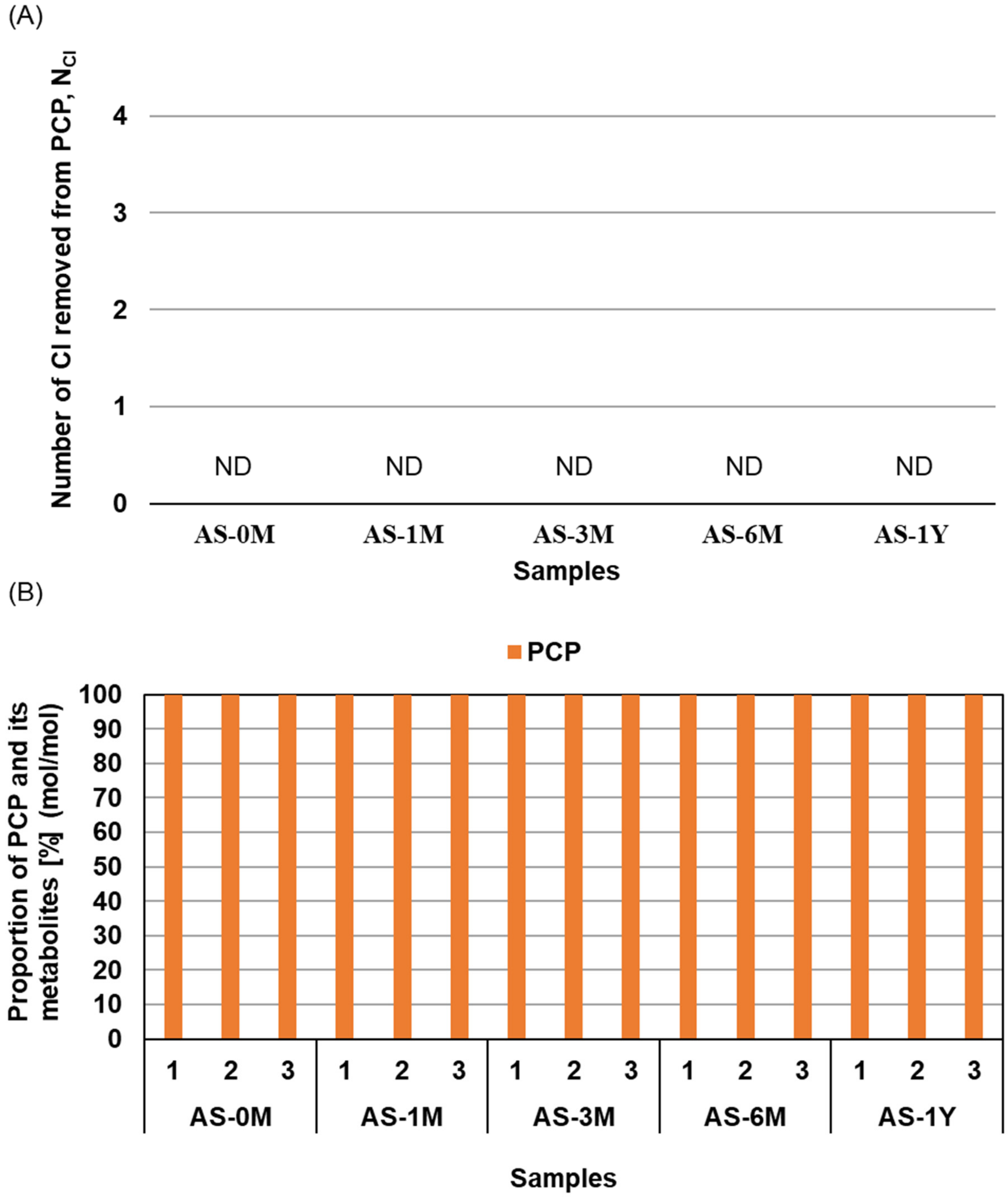

Figure S5. No EEM functionality of the artificial soil only (no rice straw and with inoculation) with 0, 1, 3, 6 months, and one year of humification (AS-0M, AS-1M, AS-3M, AS-6M, and AS-1Y), as shown by no Cl removal from PCP (A) and no dechlorination of PCP for individual samples in the EEM material-dependent PCP-dechlorinating culture (third-generation) (B). The results are summarized as negative control-b in Figure S4. ND denotes not detected.

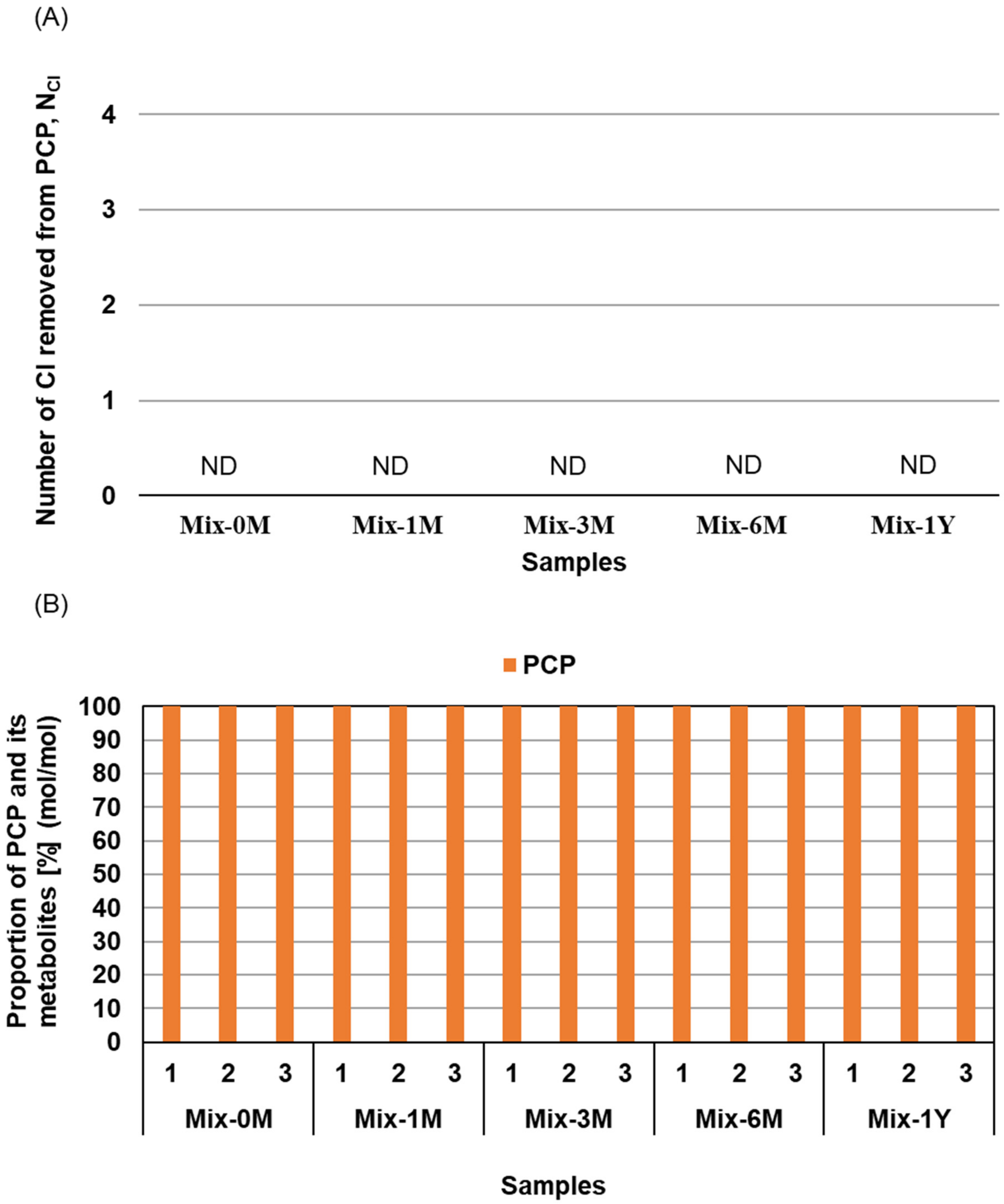

Figure S6. No dechlorination of PCP under abiotic conditions in the EEM material-dependent PCP-dechlorinating medium supplemented with the rice straw-artificial soil mixtures with 0, 1, 3, 6 months, and one year of humification (Mix-0M, Mix-1M, Mix-3M, Mix-6M, and Mix-1Y), as shown by no Cl removal from PCP (A) and proportion of PCP and its metabolites for individual sample (B). The results are summarized as negative control-a in Figure S4. ND denotes not detected.

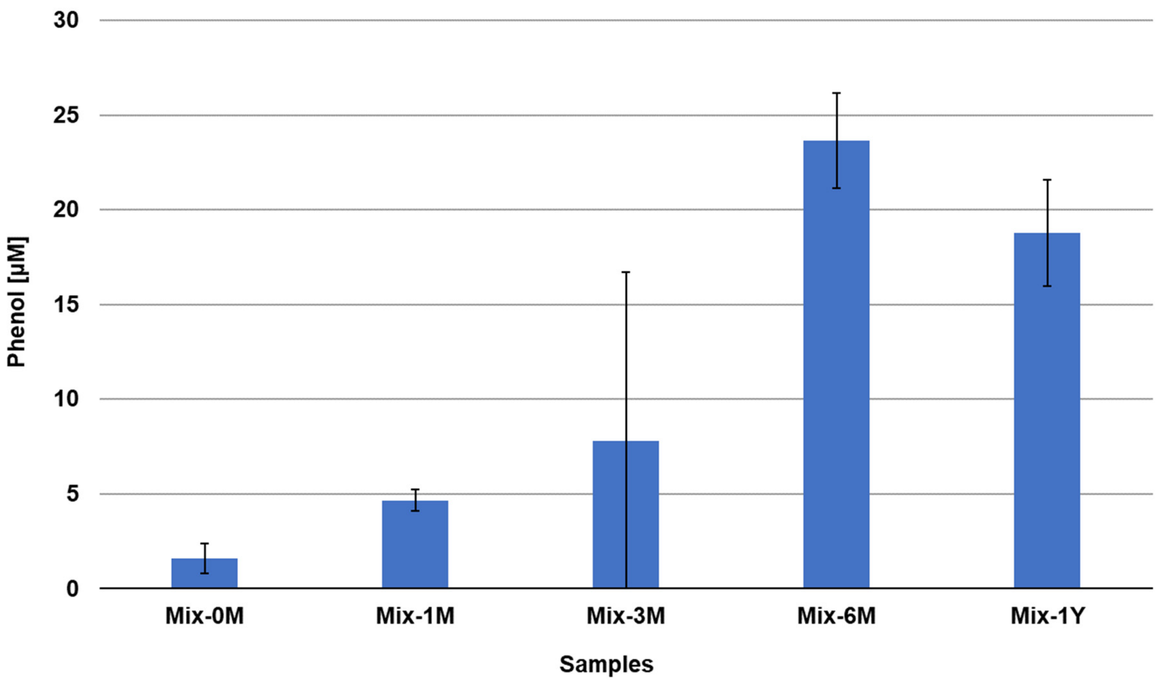

Figure S7. Changes in phenol amount in the EEM material-dependent PCP-dechlorinating culture (third-generation), supplemented with the rice straw-artificial soil mixtures with 0, 1, 3, 6 months, and one year of humification (Mix-0M, Mix-1M, Mix-3M, Mix-6M, and Mix-1Y).

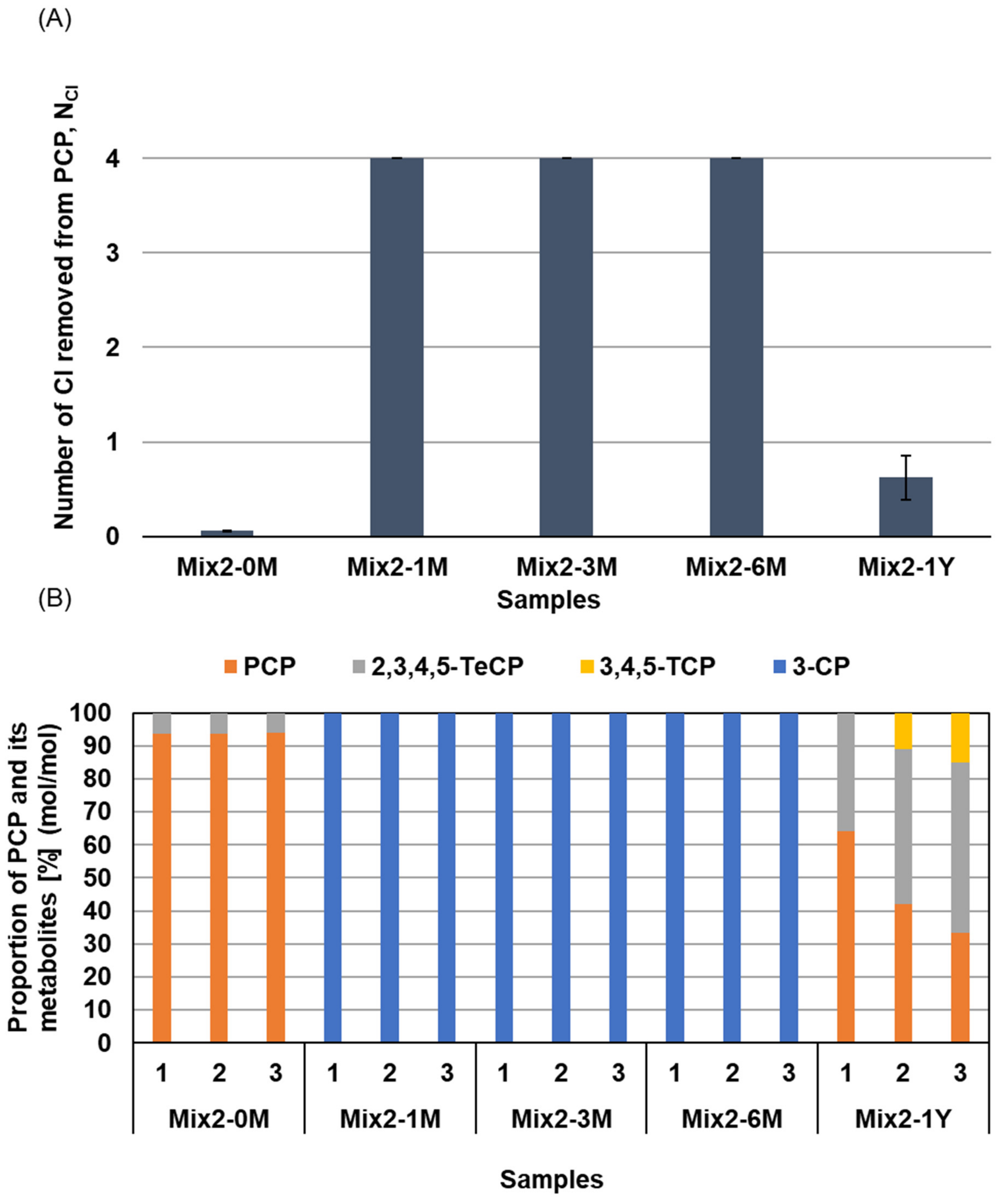

Figure S8. Changes in EEM functionality of rice straw-artificial soil mixtures with 0, 1, 3, 6 months, and one year of humification in the second experiment (named as Mix2-0M, Mix2-1M, Mix2-3M, Mix2-6M, and Mix2-1Y), as shown by number of Cl removed from PCP (A) and the PCP dechlorination activity for individual sample in the EEM material-dependent PCP dechlorinating culture (third-generation) (B).

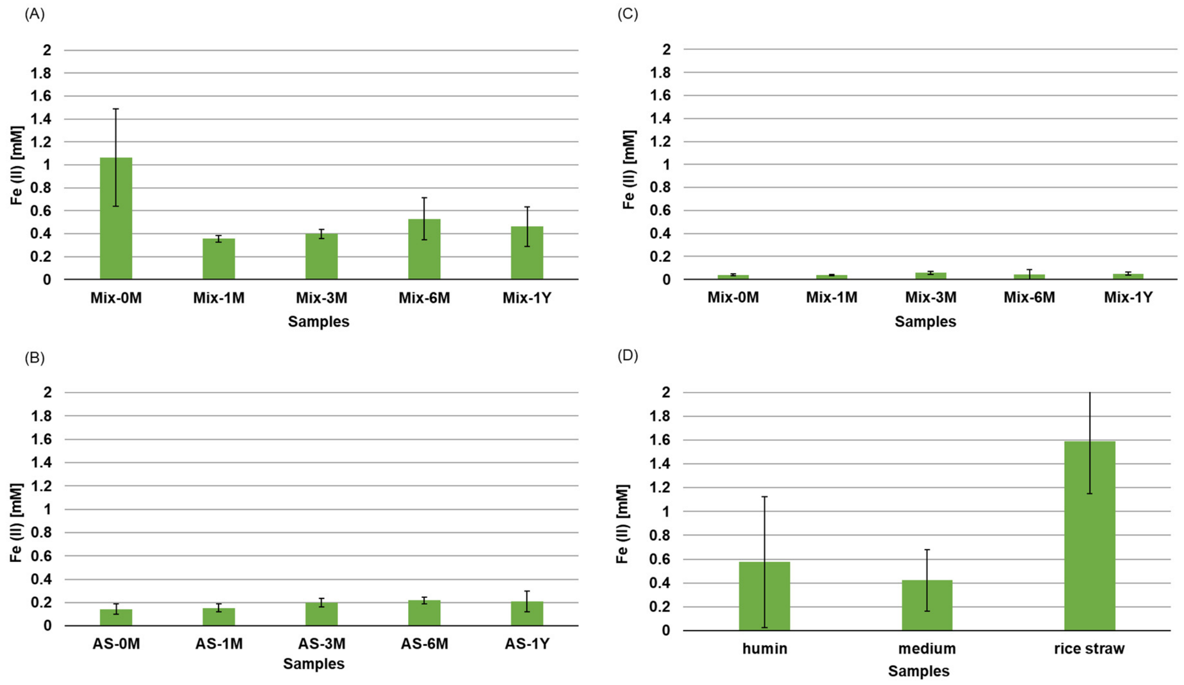

Figure S9. Dissimilatory iron reduction activity of (A): rice straw-artificial soil mixtures with 0, 1, 3, 6 months, and one year of humification (named as Mix-0M, Mix-1M, Mix-3M, Mix-6M, and Mix-1Y). (B): abiotic conditions in medium supplemented with the rice straw-artificial soil mixtures. (C): the artificial soil only (no rice straw and with inoculation) with 0, 1, 3, 6 months, and one year of humification (AS-0M, AS-1M, AS-3M, AS-6M, and AS-1Y). (D): humin (40 g/L), medium only, and rice straw (15 g/L).

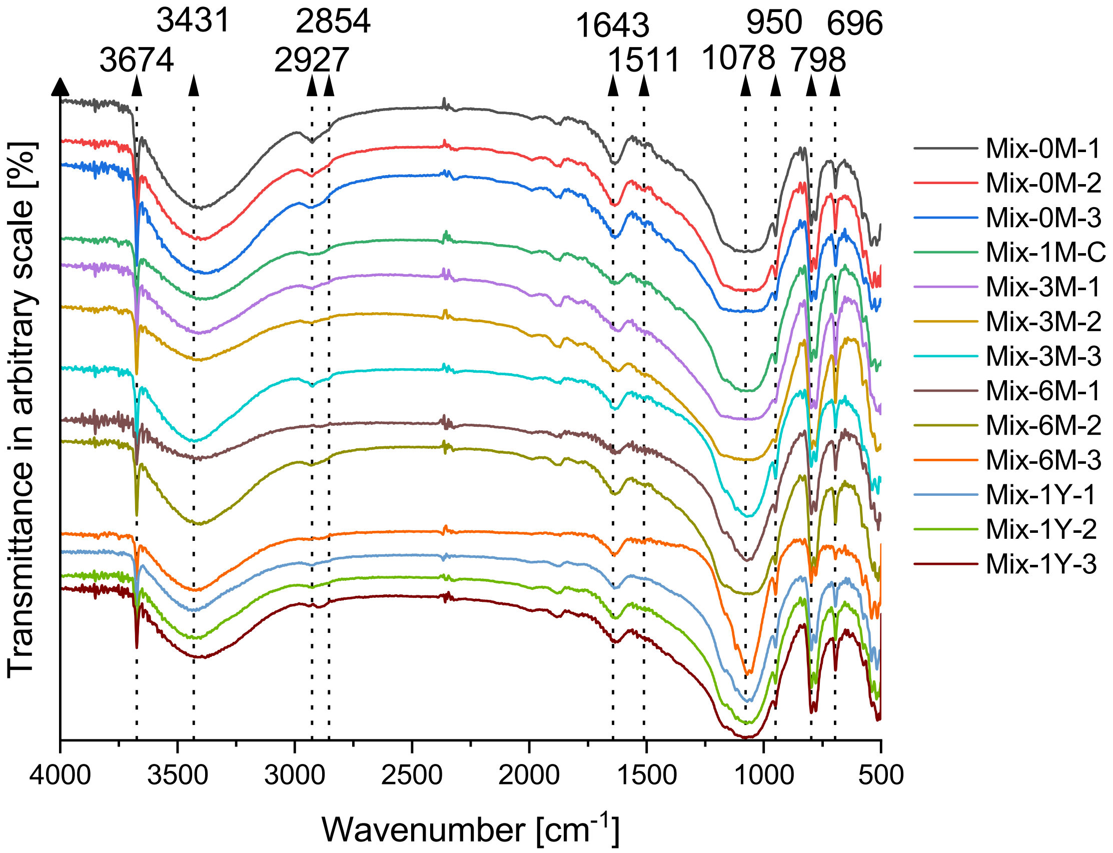

Figure S10. The FT-IR spectra of rice straw-artificial soil mixtures with 0, 1, 3, 6 months, and one year of humification. The measurement was carried out with individual sample except Mix-1M-C (composite sample).

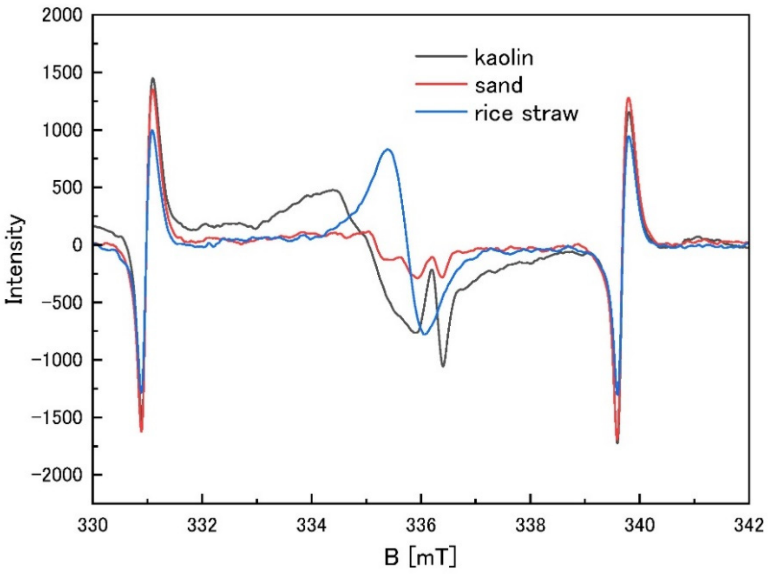

Figure S11. The ESR spectra of kaolin, sand, and rice straw with the 3rd and 4th Mn marker signals.

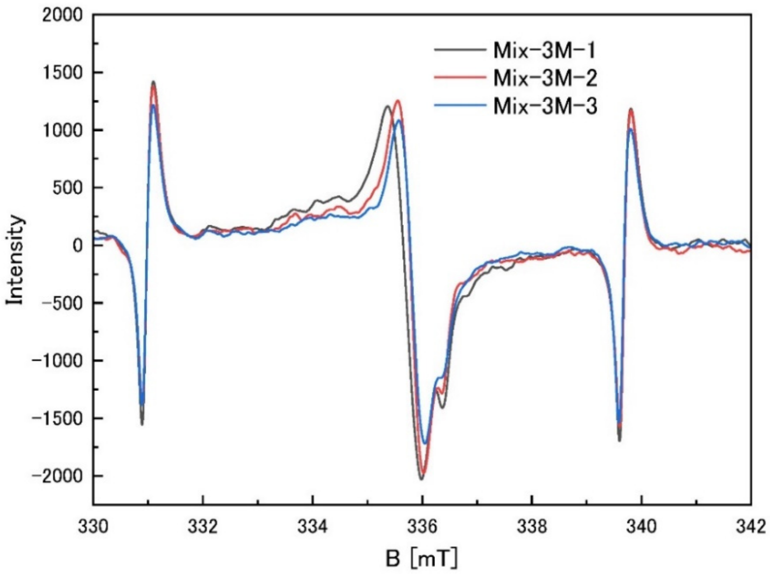

Figure S12. The ESR spectra of three replicates of rice straw-artificial soil mixtures with 3 months of humification (Mix-3M-1, Mix-3M-2, Mix-3M-3) with the 3rd and 4th Mn marker signals.

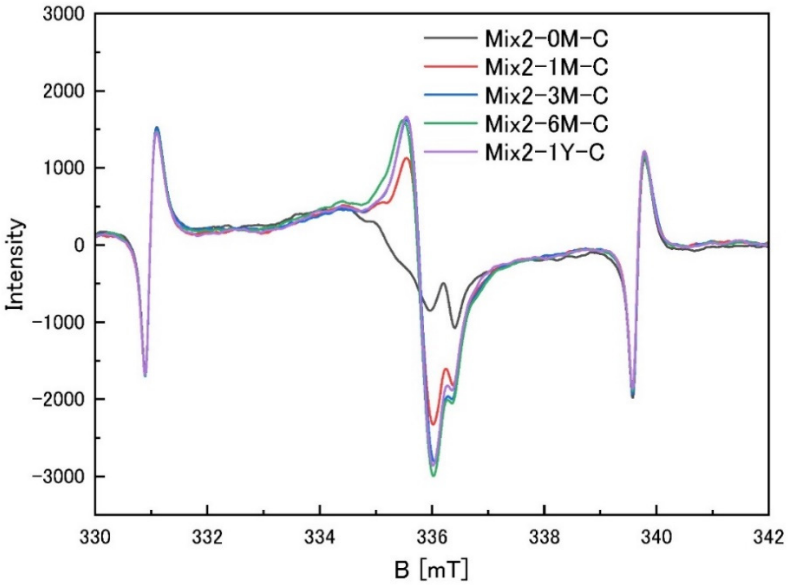

Figure S13. The ESR spectra of rice straw-artificial soil mixtures with 0, 1, 3, 6 months, and one year of humification in the second time experiment (Mix2-0M-C, Mix2-1M-C, Mix2-3M-C, Mix2-6M-C, Mix2-1Y-C) with the 3rd and 4th Mn marker signals. The measurement was carried out using the composite samples.

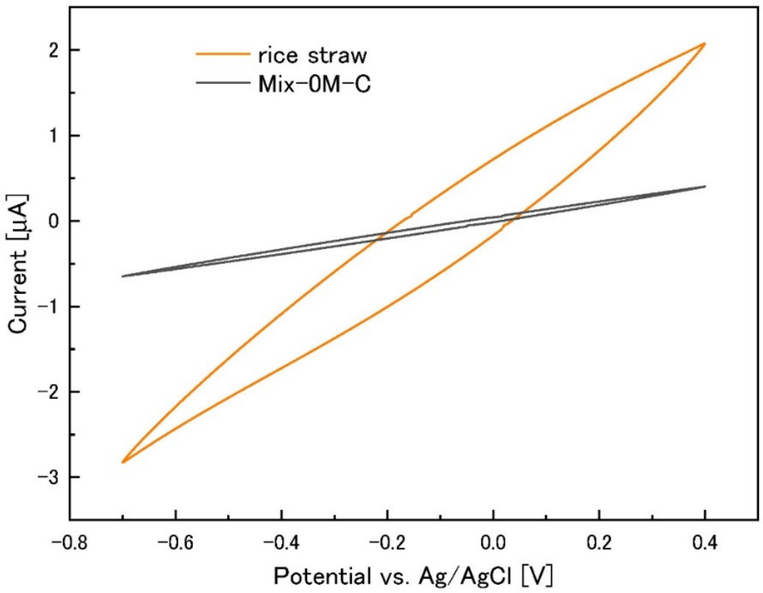

Figure S14. Cyclic voltammograms (10th cycle) of rice straw and a composite sample of 0-month rice straw-artificial soil mixture (Mix-0M-C).

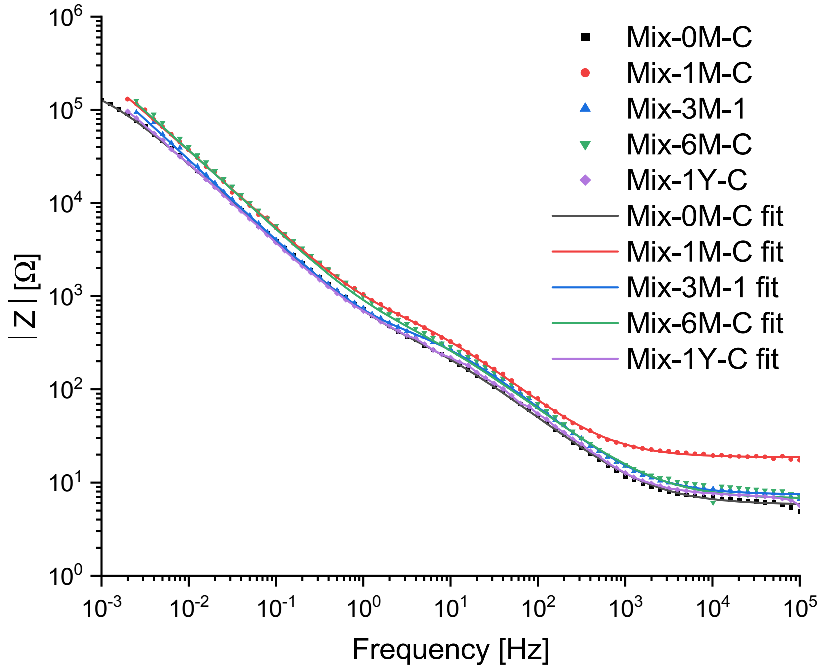

Figure S15. Electrochemical impedance spectra (EIS) (Bode plots) and fitting curves of rice straw-artificial soil mixtures with 0, 1, 3, 6 months, and one year of humification (Mix-0M-C, Mix-1M-C, Mix-3M-1, Mix-6M-C, Mix-1Y-C). The measurement was carried out using the composite samples except for Mix-3M-1.

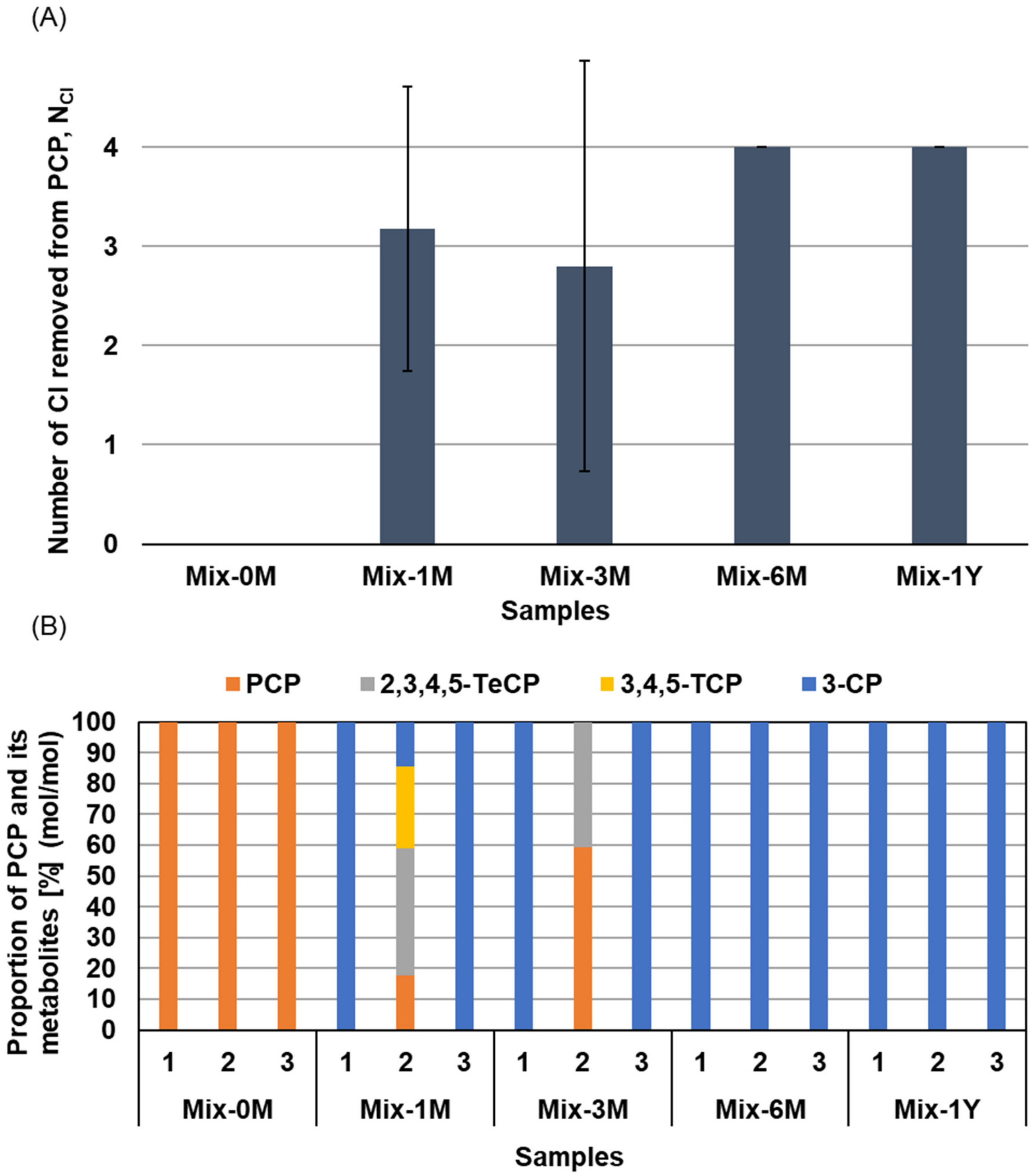

Figure S16. Dechlorination activity of EEM material-dependent PCP dechlorinating consortium in the organic carbon source-free medium (no sodium formate) amended with rice straw-artificial soil mixtures with 0, 1, 3, 6, months, and one year of humification (Mix-0M, Mix-1M, Mix-3M, Mix-6M, and Mix-1Y) (third-generation), as shown by the number of Cl removed from PCP (A) and the proportion of PCP and its metabolites for individual sample (B).

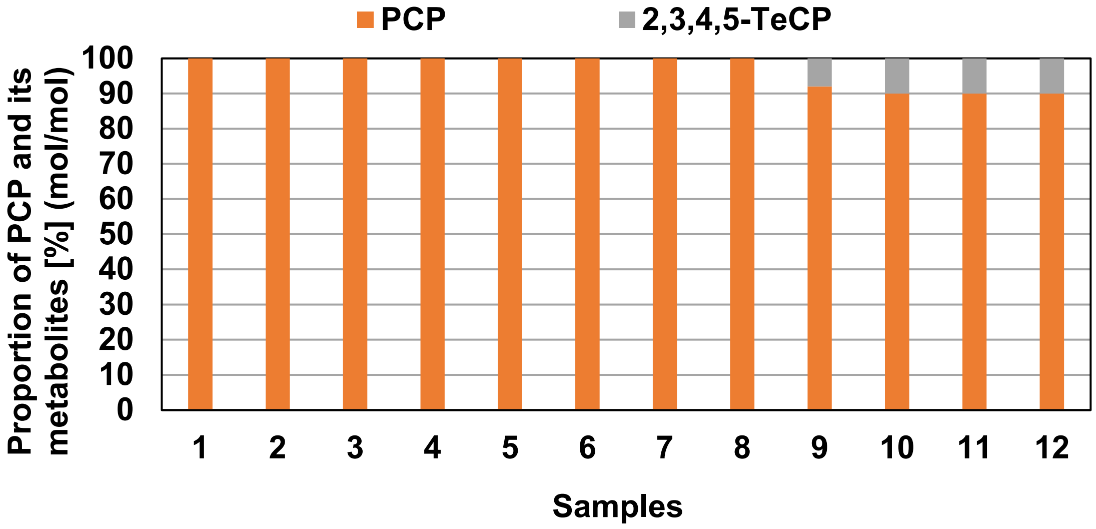

Figure S17. No EEM functionality of selected microbial biomass (freeze-dried), as shown by no dechlorination of PCP in the EEM material-dependent PCP dechlorinating culture. The samples shown by numbers are listed in the table below.

Samples	Amount [g/20mL]	Microorganisms	Type	
1	0.3	*Glycomyces algeriensis*	Prokaryote	G+
2	0.3	*Nocardioides pyridinolyticus*	Prokaryote	G+
3	0.3	*Actinomadura rubrobrunea*	Prokaryote	G+
4	0.3	*Nocardiopsis dassonvillei* subsp.*dassonvillei*	Prokaryote	G+
5	0.3	*E.coli* K-12	Prokaryote	G –
6	0.3	*Pseudomonas putida*	Prokaryote	G –
7	0.3	*Aspergillus candidus*	Eukaryote	
8	0.3	*Pleurotus ostreatus*	Eukaryote	
9	0.3	*Grifola frondosa*	Eukaryote	
10	0.3	*Lentinula edodes*	Eukaryote	
11	0.3	*Pleurotus eryngii*	Eukaryote	
12	0.3	*Flammulina velutipes*	Eukaryote	

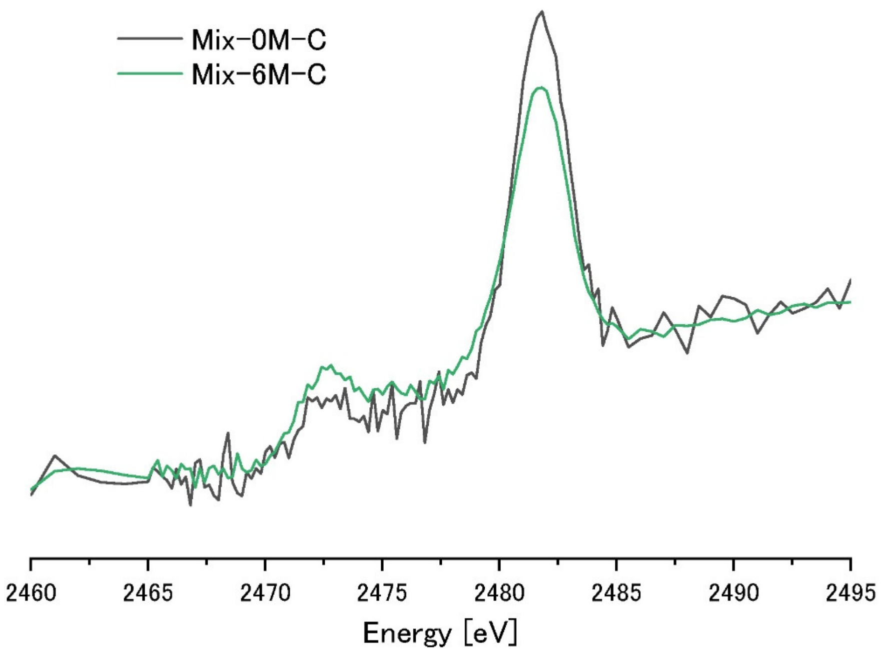

Figure S18. Sulfur K-edge X-ray absorption near-edge structure spectra of rice straw-artificial soil mixtures with 0 and 6 months of humification (Mix-0M-C, Mix-6M-C).

The measurement was carried out using the composite samples.

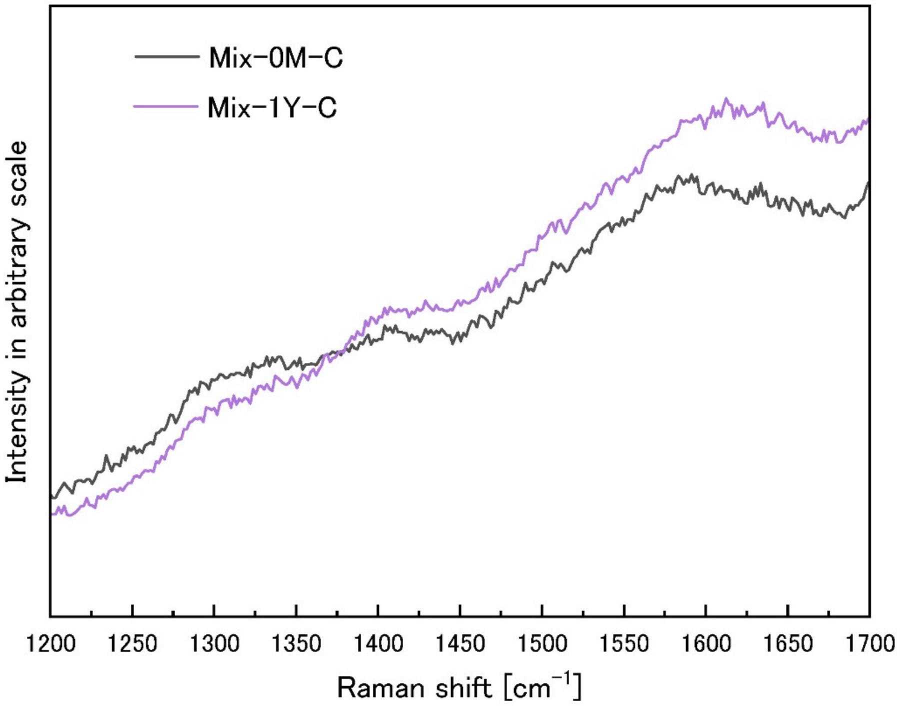

Figure S19. Raman spectra of rice straw-artificial soil mixtures with 0 month and one year of humification (Mix-0M-C, Mix-1Y-C). The measurement was carried out using the composite samples.
